# Disorder and Halide Distributions in Cesium Lead Halide Nanocrystals as Seen by Colloidal ^133^Cs Nuclear Magnetic Resonance Spectroscopy

**DOI:** 10.1021/acs.chemmater.3c02901

**Published:** 2024-03-15

**Authors:** Marcel Aebli, Christoph J. Kaul, Nuri Yazdani, Franziska Krieg, Caterina Bernasconi, Dominic Guggisberg, Malwina Marczak, Viktoriia Morad, Laura Piveteau, Maryna I. Bodnarchuk, René Verel, Vanessa Wood, Maksym V. Kovalenko

**Affiliations:** † Department of Chemistry and Applied Biosciences, ETH Zürich, Vladimir-Prelog-Weg 1-5, Zürich CH-8093, Switzerland; ‡ Empa-Swiss Federal Laboratories for Materials Science and Technology, Überlandstrasse 129, Dübendorf CH-8600, Switzerland; § Department of Information Technology and Electrical Engineering, ETH Zürich, Vladimir-Prelog-Weg 1-5, Zürich CH-8093, Switzerland

## Abstract

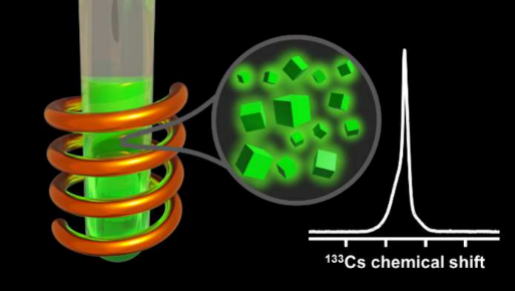

Colloidal nuclear magnetic resonance (cNMR) spectroscopy on inorganic cesium lead halide nanocrystals (CsPbX_3_ NCs) is found to serve for noninvasive characterization and quantification of disorder within these structurally soft and labile particles. In particular, we show that ^133^Cs cNMR is highly responsive to size variations from 3 to 11 nm or to altering the capping ligands on the surfaces of CsPbX_3_ NCs. Distinct ^133^Cs signals are attributed to the surface and core NC regions. Increased heterogeneous broadening of ^133^Cs signals, observed for smaller NCs as well as for long-chain zwitterionic capping ligands (phosphocholines, phosphoethanol(propanol)amine, and sulfobetaines), can be attributed to more significant surface disorder and multifaceted surfaces (truncated cubes). On the contrary, capping with dimethyldidodecylammonium bromide (DDAB) successfully reduces signal broadening owing to better surface passivation and sharper (001)-bound cuboid shape. DFT calculations on various sizes of NCs corroborate the notion that the surface disorder propagates over several octahedral layers. ^133^Cs NMR is a sensitive probe for studying halide gradients in mixed Br/Cl NCs, indicating bromide-rich surfaces and chloride-rich cores. On the contrary, mixed Br/I NCs exhibit homogeneous halide distributions.

Cesium lead halide perovskite (CsPbX_3_) semiconductor nanocrystals (NCs) are intensely pursued as novel light-emissive or light-harvesting materials for diverse optoelectronic applications^1,2^ such as LEDs,^3^ displays,^4^ lasers,^5,6^ photodetectors,^7^ solar cells,^8^ and quantum light sources.^9^ Their defect tolerance allows for bright photoluminescence (PL) with the near-unity quantum yield (QY)^10^ attained without surface-passivating shell, as typically required for traditional inorganic semiconductor NCs (CdSe, InP, or PbSe).^11^ To this day, it remains enigmatic to researchers as to why these superior optoelectronic properties seem to be mostly unaffected by structural defects and dynamics. The major drawbacks of perovskite NCs arise from structural softness and partially ionic bonding, causing incompatibility with polar solvents, facile sintering, and dynamic ligand binding.^12^ New ligand systems such as quaternary ammonium or bidentate zwitterionic ligands (phospholipids, phosphoethanol(propanol)amines, and sulfobetaines) greatly enhance the colloidal and structural integrity of CsPbX_3_ NCs,^13−15^ allowing to isolate monodisperse size series for subsequent optical studies or for use as building blocks in NCs assemblies.^16−20^ Advancements in the organic ligand coating must be matched with the ability to capture the related, usually subtle, and complex structural changes. In this sense, X-ray diffraction-based techniques are inherently blind for small NC sizes and NC surfaces as they yield the most meaningful interpretations only for structural features that are periodically translated into the atomic crystal. Nevertheless, X-ray scattering methods provide valuable insights about the structural disorder in CsPbX_3_ NCs, particularly using synchrotron radiation and complex data analysis.^21^ Recently, solid-state nuclear magnetic resonance (ssNMR)^22−26^ and nuclear quadrupole resonance (NQR)^23,27,28^ have been used to study bulk and nanocrystalline CsPbX_3_, as they see nuclei of interest without prerequisites in terms of crystallinity. Applying ssNMR, questions arise about the retention of the NC size (sintering) and surface state upon sample preparation, especially while highly concentrating and drying of the labile particles. The risk of sintering such structurally soft NCs is further aggravated under magic angle spinning (MAS at kHz rates), routinely applied in solid-state NMR. It is therefore much preferred to study NCs in their native colloidal state. Solution NMR is a commonplace method for characterizing the organic capping ligands:^29^ their chemical identification,^30^ ligand coverage,^31^ adsorption–desorption equilibria,^32^ etc. Surface-bound and especially NC-core species are hard to resolve owing to immense signal broadening due to slow dynamics. Increased molecular weight of the studied species results in a slower Brownian motion, which leads for large molecules in the nm-size range like polymers and proteins in high magnetic fields to longer longitudinal relaxation time (*T*_1_) and shorter transverse relaxation times (*T*_2_), both hampering the acquisition of NMR spectra, by increasing the delay between scans and broader signals, respectively.^33^ There is thus a general acceptance that NC inorganic core species shall be characterized with ssNMR methods only. Very scarce literature on solution NMR of semiconductor NC cores is represented by only two studies in 1988 by Steigerwald and Thayer: reported solution NMR studies on CdSe molecular clusters in the range of 1.2–3.5 nm in diameter,^34,35^ capturing a size dependence of the ^77^Se NMR chemical shifts and line shapes. No larger semiconductor NC sizes were studied to the best of our knowledge. Based on our calculations regarding the sensitivity of ^133^Cs and ^207^Pb compared to ^77^Se and ^113^Cd (see Table S3), the acquisition of colloidal CsPbBr_3_ NCs should be possible.

Herein we report that colloidal CsPbX_3_ NCs can indeed be readily studied by solution NMR, herein termed colloidal NMR (cNMR) and at practically convenient concentrations of the analyte (tens of milligrams of NCs per milliliter of colloid). Moreover, the ^133^Cs NMR signal readily responds to altering the NC core composition (monohalide and mixed halide), NC size, and NC surface state. In mixed Cl/Br NCs, a halide gradient is inferred, with surfaces being Br-rich. In contrast, Br/I NCs exhibit a homogeneous halide distribution throughout the entire inorganic core. Small NCs suffer from a longer healing length of the surface reconstruction, resulting in almost two layers of the perovskite structure to be disordered, as corroborated by density functional theory (DFT) calculations.

## Colloidal NMR on NCs

CsPbX_3_ NCs were synthesized and purified by hot-injection or room-temperature methods,^2,14^ characterized by absorption, PL, and TEM (Figure S1 and Table S4), redispersed in deuterated toluene or hexane, and transferred to a standard 5 mm o.d. NMR tube (see the Supporting Information for further details). All ^133^Cs and ^207^Pb NMR spectra were acquired on a 11.7 T instrument equipped with a PABBO solution NMR probe (Bruker Biospin) without any modifications.

**Figure 1:**
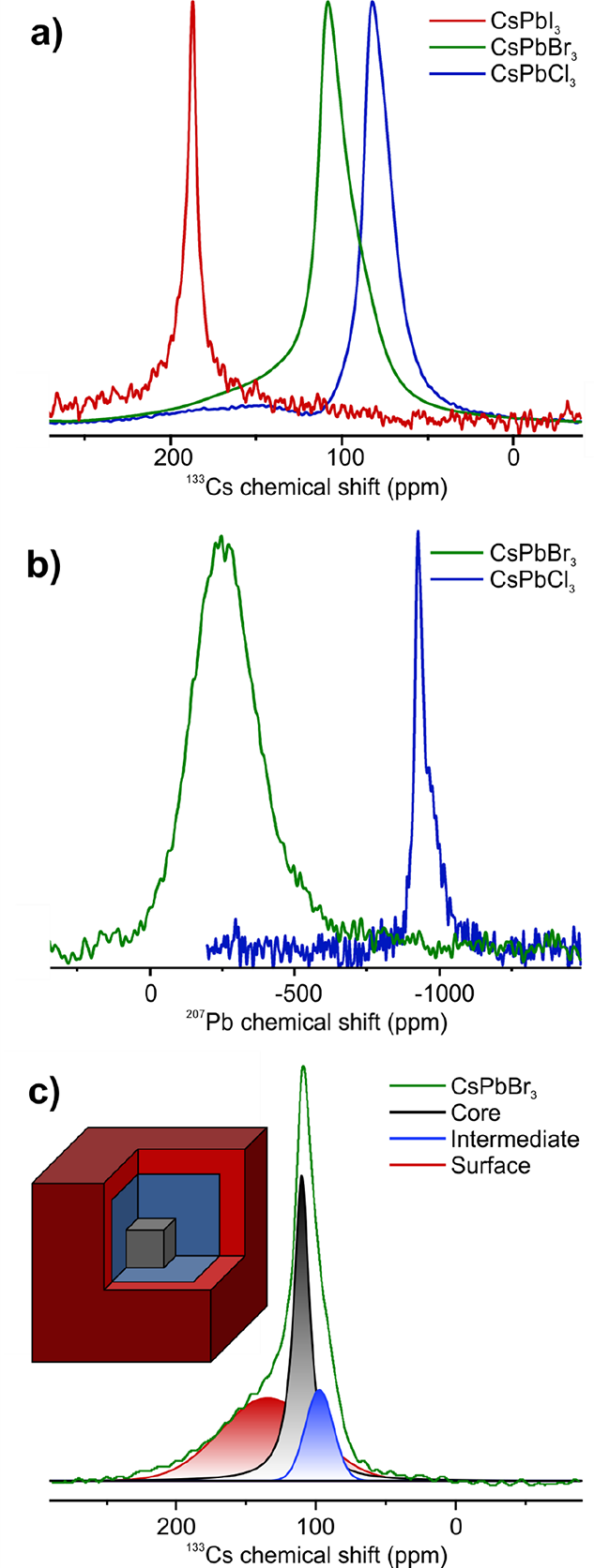
(a) ^133^Cs and (b) ^207^Pb cNMR spectra of CsPbX_3_ NCs. (c) Fitted ^133^Cs cNMR signals for core (black), intermediate (blue), and surface (red) species for a CsPbBr_3_ NC sample (green). The inset shows a scheme of a NC with the three different cesium regions.

Just like solution NMR of organic molecules, cNMR also features only isotropic chemical shifts without changes in line shapes induced by chemical shift anisotropy or spinning side bands, as often observed in ssNMR (Figure 1). cNMR spectra of CsPbX_3_ NCs (Cl, Br) of satisfying quality can be acquired in ca. 2 h for ^133^Cs (Figure 1a) and in more than a day for ^207^Pb (Figure 1b). Acquiring a ^207^Pb cNMR spectrum of CsPbI_3_ NCs was prohibited by their broader signals and structural instability (gradual conversion into a non-perovskite polymorph). We therefore focused on ^133^Cs in this study. Satisfying signal-to-noise ratios are reached at concentrations on the order of 10 mg of (CsPbX_3_)/mL, corresponding to about 10^15^ NCs (Figure S13). The largest studied particles were 17 nm sized CsPbI_3_ NCs, showcasing the large size range accessible by cNMR. The utility of ^133^Cs cNMR beyond perovskites was further demonstrated on CsBr NCs (Figures S12 and S14).

**Figure 2:**
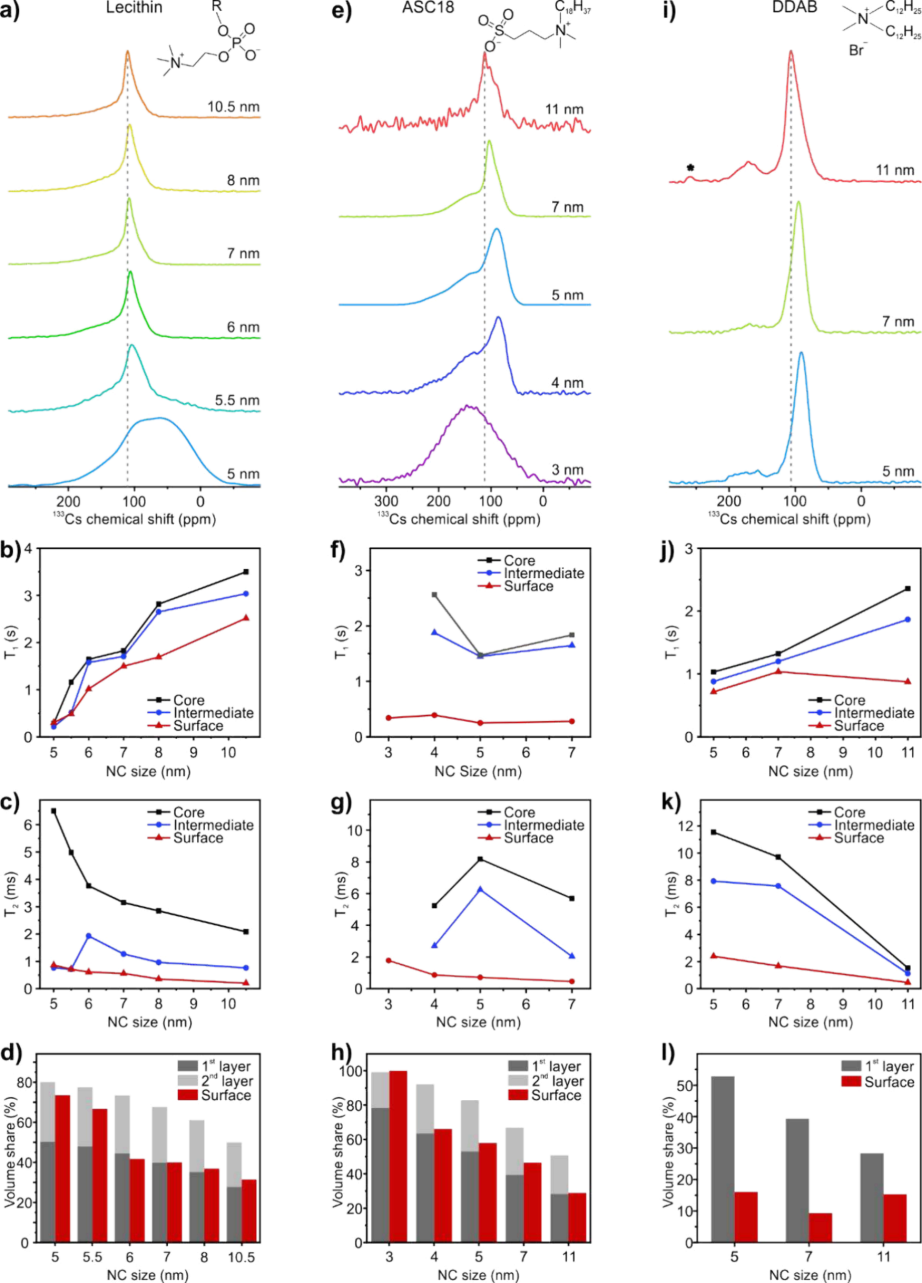
^133^Cs cNMR spectra (a, e, i), extracted *T*_1_ (b, f, j), and *T*_2_ (c, g, k) relaxation times for the fitted core, intermediate and surface species, and volume shares of the surface species compared to theoretical volumes of the first and second outer layers (d, h, l) of size selected, monodisperse CsPbBr_3_ NCs capped with lecithin (a–d), ASC18 (e−h), and DDAB (i−l). The dashed lines in (a, e, i) are guidelines to the eye, marking the chemical shift of the core signals for the biggest NCs with the corresponding capping ligand. The asterisk in (i) marks an unknown impurity.

The ^133^Cs cNMR spectra of CsPbX_3_ NCs generally show an asymmetric signal, attributed to a continuous distribution of chemical environments, analogous to ssNMR measurements of the same material.^25,26,36^ The spectra were fitted with a minimal number of Voigt peaks:^37^ two narrow peaks, corresponding to the core and an intermediate species, and one broader peak shifted to higher ppm values (Figure 1c). This broad signal was attributed to inhomogeneously broadened sites at the NC surface by cross-polarization ssNMR experiments (Figure S15). This is in agreement with previously reported experiments on CsPbBr_3_ NCs capped with monodentate ligands.^36^ The narrow signals were assigned to the central core region and an intermediate (or subsurface) species of the NC, according to the literature (see inset in Figure 1c).^25,26^ The core and intermediate signals are shifted to lower frequency from the isotropic chemical shift of the bulk measured in ssNMR, while they exhibit the same chemical surrounding. Chemical shift changes are most likely induced by lattice expansions, being more prominent in the intermediate regions, feeling a higher surface influence than the core. Further studies of the chemical shift dependence on structural variations could give a better insight into structural distortions within the NCs. The ^133^Cs chemical shifts are known to be temperature sensitive.^38^ Thus, to exclude any temperature effect induced by the MAS, bulk CsPbBr_3_ powder was measured with the same setup used for the cNMR measurements and used as a reference (Figure S16). The Brownian tumbling of the NCs in their colloidal state not only affects the shape of their ^133^Cs NMR signals but also their relaxation times. The transition from bulk CsPbBr_3_ to CsPbBr_3_ NCs in ssNMR leads to a decimation of *T*_1_, from over 100 s to 4–24 s, for 6–13 nm sized NCs, induced by a larger distribution of local environments, leading to enhanced spin–lattice relaxation.^25^ This was supported by the observation of even faster *T*_1_ relaxation times for surface or near-surface species compared to the inner core signals. In their colloidal state, the *T*_1_ relaxation of NCs is further stimulated by field fluctuation near the Larmor frequency induced by their tumbling and possible solvent interactions, reducing the *T*_1_ times to below 4 s, even for 11 nm sized particles (Figure 2b,f,j). The surface signals exhibit the shortest *T*_1_ relaxation times, confirming previous observations with ssNMR.^25^ The corresponding *T*_2_ relaxation times are 3 orders of magnitude faster, further highlighting the fact that a bulk like material is studied, because for small molecules *T*_2_ ≈ *T*_1_ (Figure 2c,g,k).^39^ The surface signals show the fastest, and the core signals show the slowest, *T*_2_* relaxation, providing a deeper insight into the increased amount of disorder located at the surface regions. Distinguishing between static and dynamic disorder would require further in-depth variable temperature (VT) experiments in a broad temperature range, naturally possible only through ssNMR, as otherwise colloids destabilize or freeze upon cooling or NCs degrade upon heating in a solvent. With cNMR at room temperature, no degradation could be observed after several days of measurements.

## Ligand and Size Dependence

The monodisperse 5–10.5 nm size series (±1 nm) of lecithin-capped CsPbBr_3_ NCs was prepared by postsynthetic size-selective precipitation (see the Supporting Information for further details and Figures S2–S3 and Table S5).^14,16^*T*_1_ relaxation times in cNMR increase monotonically with NC size. For the smallest NCs the signals strongly overlap, hampering a proper analysis. The *T*_2_ relaxation shows the opposite trend, becoming faster for bigger NCs. Both *T*_1_ and *T*_2_ correlate with the decrease in correlation time due to the slower tumbling rate for larger objects observed by solution NMR. The relaxation times in cNMR therefore follow the classical behavior of NMR of macromolecules, enabling faster acquisitions compared to ssNMR due to reduced *T*_1_ relaxation times.

The spectra of the smallest NCs are drastically different (Figure 2a,e,i). The surface species become most pronounced, while the core and intermediate species signals almost disappear. By integration of the fitted peaks, the volumetric fractions of the individual species were extracted. For NCs larger than 6 nm, the surface species takes up one monolayer of the NCs (Figure 2d), further supporting our peak assignment. Decreasing the size below 6 nm led to increased disorder. Up to two monolayers of the NCs were attributed to the disordered species, contributing more than 70% of the total volume of the particles. This showcases the high amount of surface energy in the smallest NCs, with only minimal bulk-like material in the core. Smaller NCs with the same chemical composition using lecithin as a capping ligand could not be synthesized.

**Figure 3:**
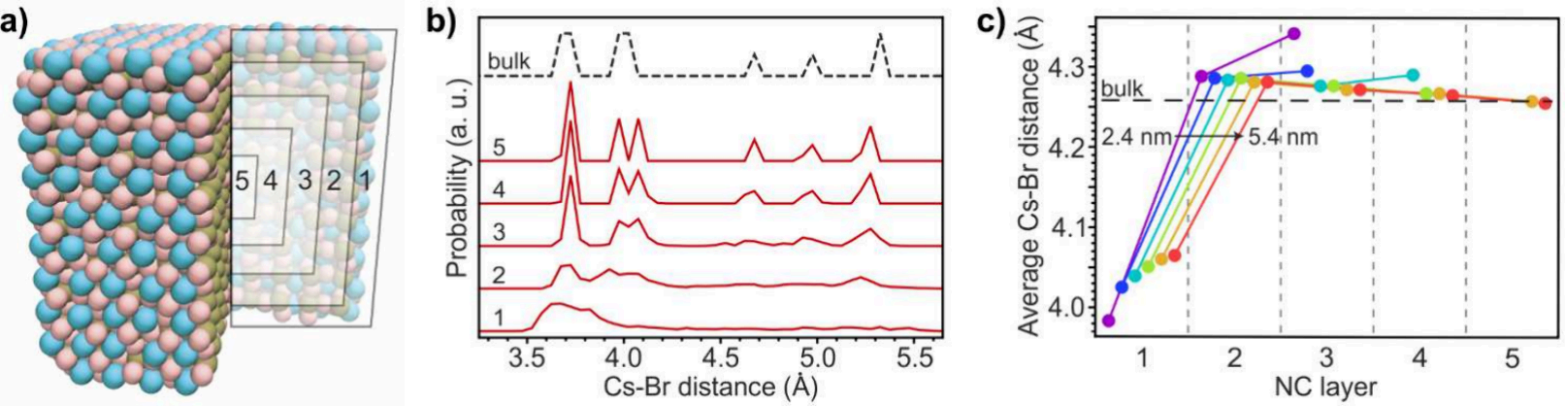
DFT calculations of CsPbBr_3_ NCs. (a) Atomistic model of a 5.4 nm CsPbBr_3_ NC with a cross section removed to indicate five regions of interest within the NC, from the outer layers (1) to the central core of the NC (5) with cesium in blue, bromide in red and lead in brown. (b) Distribution of Cs–Br distances within each region of a 5.4 nm NC. The dashed line shows the distribution of the optimized orthorhombic structure of bulk CsPbBr_3_. (c) Average Cs–Br distances in each of the regions for various sizes of NCs. The horizontal dashed line indicates the average distance for the optimized orthorhombic bulk structure of CsPbBr_3_.

To corroborate these findings, density functional theory (DFT) structure calculations were performed on CsPbBr_3_ NCs with sizes ranging from 2.4 to 5.4 nm (Figure 3). The NCs were initially cut from an optimized bulk orthorhombic lattice and then fully geometrically relaxed. Surface reconstruction leads to a distortion of the outer layers, resulting in a disordered shell around the crystalline inner core (Figure 3b). By analyzing the mean Cs–Br distances as a function of the distance from the NC surface, a strong contraction resulting from the undercoordination of surface Cs^+^ ions can be observed in the outmost layer (Figure 3c). These contractions further drive mild expansions of the Cs–Br distances deeper into the NC. The impact of the surface disorder vanishes toward the NC core, and at a depth of ca. three CsPbBr_3_ layers the bulk orthorhombic structure is intact, seen also from the Cs–Pb and Cs–Cs distances within the individual layers (Figures S20 and S21). For the smallest NCs below 3.4 nm a highly crystalline core cannot be fully stabilized. While these calculations do not account for the effect of ligands, they still corroborate the qualitative interpretation of the ^133^Cs cNMR for size-selected CsPbBr_3_ NCs and assignment of the core, intermediate, and surface regions.

Similar trends in ^133^Cs cNMR were observed for 3-(*N*,*N*-dimethyloctadecylammonio)propanesulfonate (ASC18)-capped CsPbBr_3_ NCs (Figures S4–S5 and Table S6). ASC18, as well as lecithin, is a zwitterionic ligand, binding covalently to the NC surface.^13^ This ligand affords the isolation of CsPbBr_3_ NCs as small as 3 nm. ^133^Cs cNMR for sizes down to 4 nm still shows a distinct core signal and a disordered surface covering one monolayer of the particles (Figure 2h). The 3 nm sized NCs no longer show such distinct species but only one broad signal. Didodecyldimethylammonium bromide (DDAB) is another popular ligand choice for diverse applications of lead halide perovskite NCs, ranging from LEDs to single-particle spectroscopy.^40,41^ This ligand is known to effectively passivate the surface trap states (thereby enhancing the PL QY) and stabilize the (001) facets and thus favor a sharp cuboid shape.^42^ We studied 5, 7, and 11 nm DDAB-capped CsPbBr_3_ NCs (Figures S6–S7 and Table S7) with cNMR. In contrast to the zwitterionic capped NCs, the disordered surface signal of DDAB-capped NCs is observed at 170 ppm is observed as a baseline resolved peak. Even in the smallest (5 nm) NCs the surface signal does not account for more than 16% of the particle volume (Figure 2l). A full surface monolayer at this size takes up around 50% of the surface area, indicating an overall high crystallinity and atomic surface ordering.

Across all studied ligands, one sees a pronounced size effect on the chemical shift of the NC core signal. A general trend is shifting to higher ppm for larger NC sizes, as previously observed for CdSe NCs with ssNMR.^43^ The core chemical shift of DDAB and ASC18-capped NCs changes linearly with respect to the band gap, caused by the sensitivity of the isotropic chemical shift to the band gap.^44^ The slope is ligand-dependent, highlighting the difference in surface passivation between mono- and bidentate capping ligands (Figure S17).

## Mixed Halide NCs

**Figure 4:**
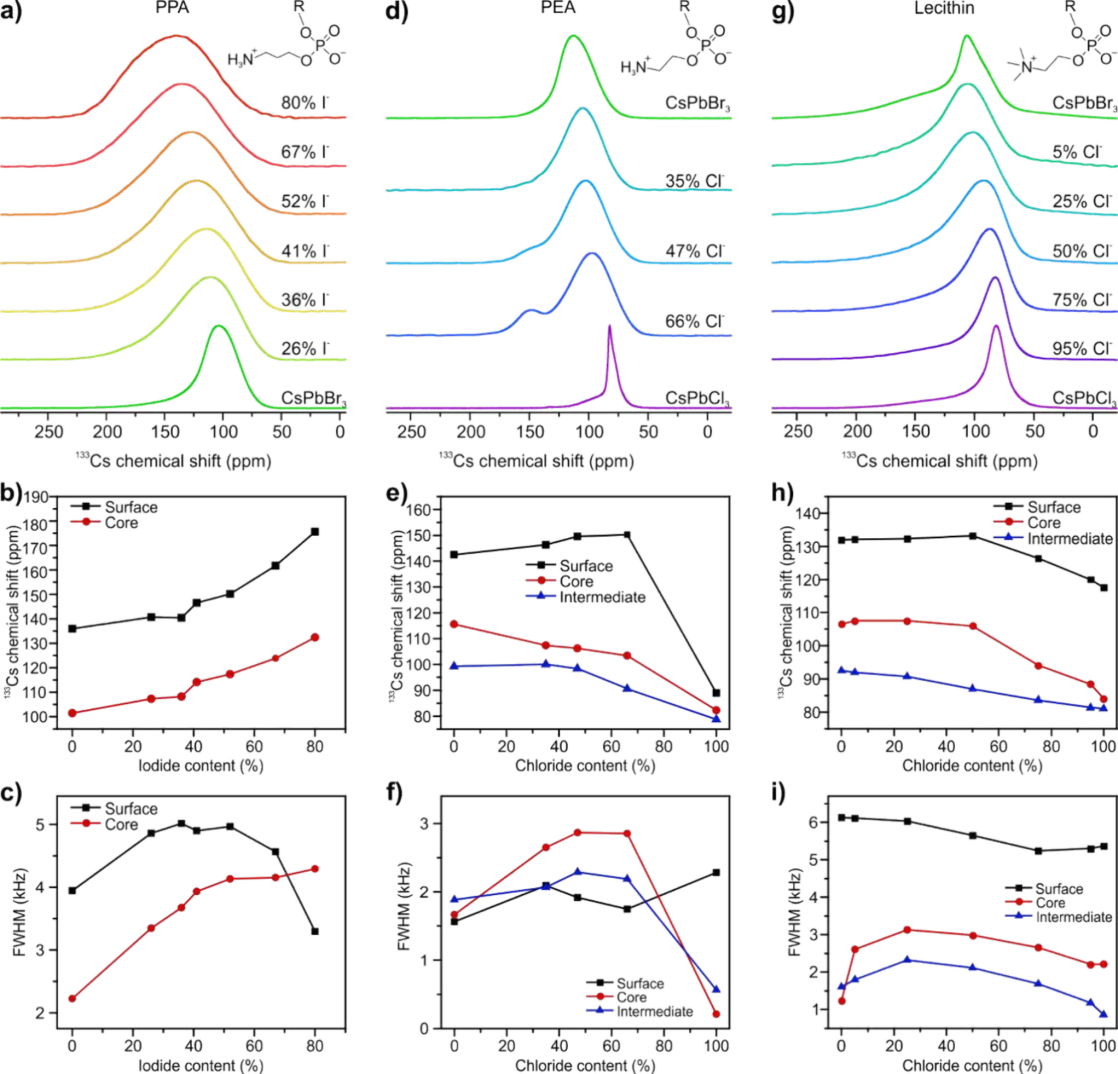
^133^Cs cNMR spectra (a, d, g), chemical shifts (b, e, h), and full widths at half-maximum (c, f, i) of the fitted surface and core peaks for PPA-capped CsPb(Br/I)_3_ (a–c) and the fitted surface, intermediate, and core peaks for PEA-capped CsPb(Br/Cl)_3_ (d–f) and lecithin-capped CsPb(Br/Cl)_3_ (g–i) NCs.

Mixed halide perovskite NCs were prepared by mixing lecithin-capped CsPbBr_3_ and CsPbCl_3_ NCs (Figures S10–S11 and Table S12), utilizing their fast anion exchange in solution,^45^ as well as by direct room temperature synthesis for Br/Cl and Br/I mixtures capped with phosphoethanolammonium (PEA, for Br/Cl) or phosphopropanolammonium (PPA, for Br/I) ligands (see the Supporting Information for further details)^15^ and were analyzed by absorption, PL, TEM, and EDX (Figures S8–S9 and Tables S7–S10). The ^133^Cs cNMR signals for Br/Cl NCs were fitted with three Voigt peaks, while for Br/I two peaks (core and surface) were already sufficient (Figures 4a,d,g and S18). The absence of an intermediate species for Br/I NCs could be caused by the overall increase of FWHM for iodine-rich NCs or an increase in dynamic or static disorder. For Br/I-NCs, both peaks shift simultaneously upon changing the halide ratios (Figure 4b), indicating a homogeneous halide distribution throughout the whole NC. The core signal broadens for higher iodide content, in good agreement with the behavior seen for the ^133^Cs line width in ssNMR on bulk mixed-halide perovskites.^46^ The surface signal is the narrowest for pure halides, while it broadens for their mixtures. Simultaneously, the integral ratio of the surface increases from around 25% for pure halides to 45% for mixed halide NCs, which corresponds to one full surface layer (Figure S19). Both factors depict increased disorder on the surface of mixed Br/I NCs compared with their pure halide analogues.

Incorporation of chloride into CsPbBr_3_ NCs does not alter the chemical shift of the surface species up to a chloride content of 50%, while the core and intermediate signals not only broaden by a factor of 2 to 3 but also shift even at lower chloride content (Figure 4e,f,h,i). This observation is seen across all Br/Cl samples, regardless of their synthesis path (direct synthesis or by anion exchange) and surface ligands. We explain this observation by the establishment of the halide gradient within the mixed Br/Cl NCs, favoring bromide-terminated surfaces and chloride-rich cores. Complementary studies such as synchrotron-based X-ray techniques applied to the same materials could give further insights into the halide distribution within the mixed-halide NCs, while taking every precaution to avoid degradation of the soft and labile NCs.^47^

## Conclusion

In summary, we showcase ^133^Cs cNMR as a simple, fast, and readily accessible (with conventional solution NMR spectrometers) tool for noninvasive characterization of CsPbX_3_ NCs in their natural colloidal state. The cNMR spectra resemble solution NMR in terms of signal shape and relaxation times. ^133^Cs cNMR is demonstrated to consistently respond to the alteration of the surface capping ligands or NC composition. The presence of the surface disorder was further supported by DFT calculations on various sizes of NCs. In mixed Br/Cl NCs, a halide gradient, favoring bromide on the surface and chloride in the core, was observed, while mixed Br/I NCs exhibit a homogeneous halide distribution. Further work will extend to comprehensively correlate the NC morphology (plates, rods, cuboids, and spheres) with the resulting ^133^Cs cNMR spectra.

## Experimental Section

Synthesis of CsPbX_3_ NCs was performed by hot injection and TOPO/DOPA synthesis methods. Detailed synthesis information is available in the Supporting Information.

### Solution NMR

Solution ^1^H, ^31^P, ^133^Cs, and ^207^Pb NMR spectra were recorded on a 11.7 T Bruker Avance IIIHD spectrometer (Bruker Biospin, Fällanden, Switzerland). The instrument was equipped with a BBFO-Z probe. The Larmor frequencies for ^1^H, ^31^P, ^133^Cs, and ^207^Pb were set to 500.3, 202.5, 65.68, and 104.7 MHz. The sample temperature was set to 298 K. ^1^H spectra were acquired by using a one-pulse sequence with a 3.8 μs excitation pulse and a 1 s recycle delay. ^31^P spectra were acquired by using a 30° excitation pulse (4.7 μs) and proton decoupling with a 2 s recycle delay. ^133^Cs spectra were acquired using an echo sequence to suppress pulse artifacts with a 90° excitation pulse (15.4 μs), an echo delay of 6.9 μs, and a 5 s recycle delay. *T*_1_ relaxation times were determined with a saturation recovery pulse sequence of 16 saturation pulses. *T*_2_ relaxation times were determined with a cpmg sequence.^48,49^^207^Pb spectra were acquired using an echo sequence to suppress pulse artifacts with a 90° excitation pulse (10.5 μs), an echo delay of 24.6 μs, and a 1 s recycle delay. All spectra were referenced externally to tetramethylsilane (^1^H), 85% H_3_PO_4_ in H_2_O (^31^P), 0.1 M CsNO_3_ in H_2_O (^133^Cs), and tetramethyllead (^207^Pb).

### Solid-State NMR

Solid-state ^1^H–^133^Cs NMR spectra were recorded on a 16.4 T Bruker Avance IIIHD spectrometer (Bruker Biospin, Fällanden, Switzerland). The instrument was equipped with a 2.5 mm triple-resonance MAS probe. The spectral frequency was set to 91.8 MHz for the ^133^Cs. The sample temperature was set to 298 K. ^1^H–^133^Cs spectra were acquired using a cp transfer sequence with a 90° proton excitation pulse (7 μs), a power ramp with variable contact time (2000–9000 μs), a power of 75 W, and a recycle delay of 1 s.

### Density Functional Theory (DFT) Calculations

DFT calculations were performed using the quickstep module of the CP2K program suite.^50^ A dual basis of localized Gaussians and plane waves,^51^ with a 300 Ry plane-wave cutoff, were used for the calculations, along with double-zeta-valence-polarization^52^ and Goedecker–Teter–Hutter pseudopotentials^53^ for core electrons. Because of the large system sizes considered, the PBE exchange correlation functional was employed, and spin–orbit coupling was not included. Nonperiodic boundary conditions in atomic coordinates and electric potential were used for the NC calculations, using the wavelet Poisson solver.^54^ For self-consistent-field calculations, a convergence to 10^–8^ was enforced. Geometry optimization was performed with the Quickstep module using the Broyden–Fletcher–Goldfarb–Shannon (BFGS) optimizer. Using convergence criteria of a maximum force of 24 meV Å^–1^, all atoms of the NCs were relaxed. Cell optimizations of the bulk orthorhombic unit cell were performed with a convergence to 100 bar. We include the charge compensation from the ligands in the calculations, while not explicitly including the ligands themselves.

### Absorption/PL

Optical absorption UV–vis absorption spectra for colloidal solutions were collected by using a Jasco V670 spectrometer in transmission mode. A Fluorolog iHR 320 Horiba Jobin Yvon spectrofluorometer equipped with a PMT detector was used to acquire steady-state PL spectra from the solutions.

### TEM

TEM images were collected using a Hitachi HT7700 microscope operated at 100 kV and a JEOL JEM-2200FS microscope operated at 200 kV.

### Powder X-ray Diffraction (XRD)

XRD patterns were collected in transmission mode with a STADI P diffractometer (STOE&Cie GmbH), equipped with a curved Ge (111) monochromator (Cu Kα_1_ = 1.54056 Å) and a silicon strip MYTHEN 1K detector (Fa. DECTRIS). For the measurement, ground powder was placed between the adhesive tape.

### EDX

The energy-dispersive X-ray measurements were conducted on the FEI QUANTA 200F microscope or the FEI MAGELLAN 400 microscope, both equipped with an energy-dispersive X-ray spectrometer EDAX OCTANE SUPER. The EDX measurements were performed with an acceleration voltage *U*_acc_ of 30 kV. The employed software to control the spectrometer was the software environment AMETEK-EDAX GENESIS.

## Supporting Information Available

Materials, synthesis procedure, Figures S1–S21, and Tables S1–S13 (PDF)
