# Supplementary material for: Disorder and Halide Distributions in Cesium Lead Halide Nanocrystals as Seen by Colloidal 133Cs Nuclear Magnetic Resonance Spectroscopy
Source: Chem Mater. 2024 Mar 15;36(6):2767–75. doi: 10.1021/acs.chemmater.3c02901 (PMC10976639; doi:10.1021/acs.chemmater.3c02901)
Supplement: Supplementary file 1 — cm3c02901_si_001.pdf [file cm3c02901_si_001.pdf]

Supplementary Information for

Disorder and Halide Distributions in Cesium Lead Halide Nanocrystals as Seen by Colloidal ^{133}Cs Nuclear Magnetic Resonance Spectroscopy

Marcel Aebli,[a,b] Christoph J. Kaul,[a,b] Nuri Yazdani,[c] Franziska Krieg,[a,b] Caterina Bernasconi,[a,b] Dominic Guggisberg,[a,b] Malwina Marczak,[a,b] Viktoriia Morad,[a,b] Laura Piveteau,[a,b] Maryna I. Bodnarchuk,[a,b] René Verel,[a] Vanessa Wood,[c] Maksym V. Kovalenko*,[a,b]

[a] Department of Chemistry and Applied Biosciences, ETH Zürich, Vladimir-Prelog-Weg 1-5, CH-8093, Switzerland.
[b] Empa-Swiss Federal Laboratories for Materials Science and Technology, Dübendorf, Überlandstrasse 129, CH-8600, Switzerland.
[c] Department of Information Technology and Electrical Engineering, ETH Zürich, Vladimir-Prelog-Weg 1-5, Zürich CH-8092, Switzerland.

E-mail: mvkovalenko@ethz.ch

Table of Contents

Experimental Section 2
Sample Characterizations 7
Additional NMR data 19
Additional DFT Figures 24
References 25

Experimental Section

Chemicals

Acetone (HPLC grade, Fischer), Bromine (Br_2, 99.0%, Sigma Aldrich), cesium carbonate (Cs_2CO_3, Sigma Aldrich), diethylether (Et_2O (>99.8%, Sigma Aldrich), didodecyldimethylammonium bromide (DDAB, Sigma Aldrich), ethanol (EtOH, >99.8%, Sigma Aldrich), ethylacetate (EtOAc, HPLC grade, Fischer), hydroiodic acid (HI, 57% in water, Sigma Aldrich), lead acetate trihydrate ($Pb(OAc)_2 \cdot 3H_2O$, ≥99.99%, Sigma Aldrich), lead(II) bromide ($PbBr_2$, 98%, Sigma Aldrich), mesitylene (98%, Sigma Aldrich), 1-octadecene (ODE, techn. grade 90%, Sigma Aldrich), oleic acid (OA, ≥99%, Sigma Aldrich), potassium permanganate ($KMnO_4$, Fluka), 3-(N,N-dimethyloctadecyl-ammonio)butanesulfonate (ASC18, synthesized acc. to reference)[1], soy-lecithin (Roth), toluene (puriss, Sigma Aldrich), toluene-d_8 (99.5+ atom% D, Sigma Aldrich), 2-octyldodecyl phosphoethanolamine (PEA, synthesized acc. to Ref.[2]), 2-octyldodecyl phosphopropanolamine (PPA, synthesized acc. to Ref.[2]), trioctylphosphine (TOP, min. 97%, Strem), Oleylamine (OLA, >95%, STREM), zinc bromide ($ZnBr_2$, puriss., anhydrous, ≥98%, Sigma Aldrich), zinc chloride ($ZnCl_2$, puriss., Sigma Aldrich), zinc iodide (ZnI_2, ≥98%, Sigma Aldrich), diisooctylphosphinic acid (DOPA, technical ≈ 90%, Sigma Aldrich), trioctylphosphine oxide (TOPO, 99 %, Strem Chemicals), *n*-octane (for synthesis≥99 %, Carl Roth), and *n*-hexane (suitable for HPLC ≥ 97.0 %, Sigma Aldrich). For NC purification in the glove box ultra-dry solvents were used. Toluene was dried in a molecular-sieves-based solvent drying system to achieve water content <5 ppm and then stored in a nitrogen filled glove box over molecular sieves. Ultra-dry polar acetone was delivered from Acros. Chemicals were used as provided by the manufacturer, without further purification.

Synthesis

CsOA 0.4 M in ODE. Cs_2CO_3 (1.6 g, 5 mmol), OA (5 mL, 16 mmol) and ODE (20 mL) were evacuated upon heating to 120 ºC until the completion of gas evolution. The CsOA solution was stored under argon and must be heated before use as it is solid at room temperature.

CsOA 0.02 M in *n*-hexane. Cs_2CO_3 (78.2 mg, 0.24 mmol) and OA (4 mL, 12.67 mmol) were dissolved in 20 mL of *n*-hexane at room temperature in air.

CsDOPA 0.02 M in *n*-hexane. Cs_2CO_3 (97.8 mg, 0.30 mmol) and DOPA (1 mL, 3.15 mmol) were dissolved in 2 mL of *n*-octane at 120 ºC on a hotplate in air. The reaction mixture is allowed to cool to room temperature and then diluted with 27 mL of *n*-hexane.

$Pb(OA)_2$ 0.5 M in ODE. $Pb(OAc)_2 \cdot 3H_2O$ (4.6g, 12 mmol), OA (7.6 mL, 24 mmol) and ODE (16.4 mL) were mixed in a three-necked flask and evacuated upon heating to 120 °C until the complete evaporation of acetic acid and water. The $Pb(OA)_2$ solution was stored under argon and must be heated before use as it solidifies at room temperature.

$Pb(DOPA)_2$ 0.10 M in ODE. $Pb(OAc)_2 \cdot 3H_2O$ (2.2 mmol) and DOPA (6.3 mmol) were dissolved in 20 mL of ODE and degassed in vacuo at room temperature. After the first degas, the reaction mixture was heated under vacuum to 120 ºC and degassed for one hour.

$PbBr_2$, $ZnCl_2$, $ZnBr_2$, ZnI_2, and TOPO stock solutions 0.067 M in *n*-octane. $PbBr_2$, $ZnCl_2$, $ZnBr_2$ or ZnI_2 (2.00 mmol), and TOPO (11.11 mmol) were dissolved in 25 mL of

n-octane at 120 °C in air. The TOPO solution was synthesized analogously without addition of metal halides.

TOP-Cl_2 0.5 M in toluene. TOP (6 mL, 13 mmol) with approximatively 130 mmol Cl_2 gas, which was produced by slowly adding HCl (15 mL, 170 mmol) in aqueous solution to the excess of $KMnO_4$ under argon flow. The insertion of a water-filter flask guaranteed the complete removal of traces of HCl. No vacuum grease but Teflon® joints and only equipment made of glass were used in order to avoid chlorine glass leaks. $TOPCl_2$ is solid at 0 °C and liquid at room temperature. The final stock solution was obtained by adding toluene (18.7 mL).

TOP-Br_2 0.5 M in toluene. TOP (6 mL, 13 mmol) was mixed with toluene (18.7 mL) and Br_2 (0.6 mL, 11.5 mmol) under inert atmosphere.

Oleylammonium iodide (OLAI). OLA (62.5 mL, 0.19 mol) and HI (21.5 mL, 0.19 mol) were mixed in EtOH (500 mL) and purified by recrystallization from Et_2O and EtOH.

Ligand stock solution (0.1 mg/µL) in mesitylene. The respective ligand (PEA or PPA) was dissolved in mesitylene up to the desired concentration.

Lecithin-capped $CsPbX_3$ (X = Br, Cl) NCs.[3] For $CsPbBr_3$ NCs, CsOA in ODE (4 mL, 1.6 mmol), $Pb(OA)_2$ (5 mL, 2.5 mmol) and lecithin (0.324 g, ca. 0.45 mmol) were dissolved in ODE (10 mL) and heated under vacuum to 100 ºC, where upon the atmosphere was changed to argon and TOP-Br_2 in toluene (5 mL, 2.5 mmol, 5 mmol of Br) was injected. The reaction was cooled immediately by an ice bath. For $CsPbCl_3$ NCs, CsOA (4 mL, 1.6 mmol), $Pb(OA)_2$ (5 mL, 2.5 mmol) and lecithin (0.641 g, ca. 0.90 mmol) were dissolved in ODE (5 mL) and heated under vacuum to 150 ºC, whereupon the atmosphere was changed to argon and TOP-Cl_2 in toluene (5 mL, 2.5 mmol, 5 mmol of Cl) was injected. The reaction was cooled immediately by an ice bath.

Isolation and purification. The crude solution was precipitated by the addition of 2 volumetric equivalents of acetone, followed by the centrifugation at 29500g (g is the earth gravity) for 10 minutes. The precipitated fraction was dispersed in 10 mL of toluene and then washed three more times. Each time the solution was mixed with two volumetric equivalents of acetone and centrifuged at 29500 g for 1 minute, and subsequently dispersed in the progressively smaller amounts of the solvent (5 mL for the second cycle, 2.5 mL for the third cycle). After the last precipitation, NCs were dispersed in 2 mL of toluene and centrifuged at 29500 g for 1 minute to remove any non-dispersed residue.

ASC18-capped $CsPbBr_3$ NCs.[1] ASC18 (0.216 g) was added into a 25 mL three-neck flask along with CsOA in ODE (0.4 mL, 0.16 mmol), $Pb(OA)_2$ (0.5 mL, 0.25 mmol, warm) and 5 mL ODE. The reaction vessel was purged 3 times and heated under inert gas to 130 ºC, followed by the injection of TOP-Br_2 (0.5 mL, 0.5 mmol). The reaction was immediate, and the resulting crude solution was cooled to room temperature using a water-ice bath.

Isolation (washing step 1): The crude solution was centrifuged at 29464 g for 10 minutes. The supernatant was isolated and mixed with 12 mL of ethyl acetate and the mixture was centrifuged at 29464 g for 10 minutes. The precipitate was redispersed in 3 mL of toluene. Purification (washing steps 2-4): the colloid can be further purified by up to 3 rounds of precipitation and redispersion, each comprising sequential addition of 6 mL ethyl acetate, centrifugation at 29500 g for 1 minute and subsequent redispersion in 3 mL of toluene. The final NC dispersion can be centrifuged at 29500 g for 1 minute again to remove larger NCs.

DDAB-capped $CsPbBr_3$ NCs.[4] 55 mg of $PbBr_2$ and 5 mL of ODE were added into a three-neck flask and heated to 180 °C under vacuum. At 120 ºC the atmosphere was changed to argon and 0.5 mL of dried OA along with 0.5 mL of dried OLA were injected. At 180 ºC, 0.8 mL of 0.125 M CsOA was injected into the reaction flask. After 15 s, the reaction was quenched with ice-bath. The crude solution was centrifuged at 12000 g for 3 minutes. Subsequently, the supernatant was discarded, while the precipitate was redissolved in 0.3 mL of hexane and centrifuged at 12000g for 3 minutes. After centrifugation, the precipitate was discarded and 600 µL of toluene along with 150 µL of 0.1M DDAB/$PbBr_2$ were added to the supernatant. Solution was centrifuged at 12000 g for 1 hour. Subsequently, 1.8 mL of ethyl acetate was added as antisolvent, and the solution was centrifuged at 12000 g for 3 minutes. The precipitate was redispersed in 600 µL of toluene, followed by the addition of 3 mL of ethyl acetate and centrifugation for 5 minutes at 4400 g. The supernatant was discarded, and the precipitate was redispersed in 600 µL toluene-d_8.

PEA- and PPA-capped $CsPb(Br/Cl)_3$ and $CsPb(Br/I)_3$ NCs.[2] For each cesium lead halide composition, Table S1 specifies precursors and their quantities, as well as reaction conditions. In general, lead precursor ($PbBr_2$ for $CsPb(Br/Cl)_3$ and $CsPb(Br/I)_3$, $Pb(DOPA)_2$ for $CsPbCl_3$) and zinc halide precursor(s) ($ZnCl_2$, $ZnBr_2$ and/or ZnI_2, used as convenient halide sources, *e.g.* $PbCl_2$ does not dissolve in n-octane with TOPO) were placed in one reaction vial and diluted with *n*-hexane. The reaction mixture was vigorously stirred at room temperature, followed by the swift injection of the cesium precursor (CsDOPA or CsOA), which initiated the reaction. In general, CsDOPA was used for $CsPb(Cl/Br)_3$ NCs, whereas iodide containing NCs were synthesized with CsOA. $CsPbBr_3$ NCs were possible to synthesize with both precursors. The reaction was quenched with the addition of the 0.1 mg/µL stock solution of ligand (C8C12 PEA, for $CsPb(Cl/Br)_3$ or C8C12 PPA, for $CsPb(Br/I)_3$) after the particles reached the desired size. The reaction mixture was stirred for one additional minute after ligand addition. For purification of the NCs, one to two equivalents of washing solution (EtOAc:ACN,2:1 v:v, dried over molecular sieves and filtered through 0.2 µL PTFE filter) were added to the crude reaction mixture to cause NCs precipitation, followed by centrifugation. The supernatant was discarded, and precipitate was redispersed in a minimal amount of *n*-hexane. The NCs were washed three times.

Table S1. Direct room temperature synthesis of $CsPb(Br/Cl)_3$ and $CsPb(Br/I)_3$. a) 16 mL of TOPO was added additionally. b) $PbDOPA_2$ was used for pure $CsPbCl_3$ NCs.

Sample	PL / nm	ZnI_2 TOPO / mL	$ZnBr_2$ TOPO / mL	$ZnCl_2$ TOPO / mL	$PbBr_2$ TOPO / mL	CsOA / mL	CsDOPA / mL	*n*-hexane / mL	Ligand	amount of ligand / mL	injection of ligand / s
100% Cl^-	409	-	-	8$^{a)}$	3,48$^{b)}$	-	6	5	PEA	0,4	600
66% Cl^-	452	-	-	4	5,2	-	6	20	PEA	0,4	360
47% Cl^-	470	-	-	2	5,2	-	6	20	PEA	0,4	360
35% Cl^-	483	-	-	1,2	5,2	-	6	20	PEA	0,4	360
100% Br^-	509	-	-	-	5,2	-	6	20	PEA	0,4	60
100% Br^-	503	-	5	-	2	6	-	80	PPA	1	420
26% I^-	542	2	3	-	2	6	-	80	PPA	1	183
37% I^-	553	2,5	2,5	-	2	6	-	80	PPA	1	166
41% I^-	581	3	2	-	2	6	-	80	PPA	1	149
52% I^-	600	3,5	1,5	-	2	6	-	80	PPA	1	129

67% I^-	625	4	1	-	2	6	-	80	PPA	1	108
80% I^-	642	5	-	-	2	6	-	80	PPA	1	40

CsBr NCs. CsBr NC synthesis was adapted from the literature.[5] $ZnBr_2$ (90 mg, 0.04 mmol) was dissolved in 2 mL ODE, 1 mL OA and 1 mL OLA at 75 °C. 0.5 mL of CsOA (0.4 M) was added. After 5 minutes, the reaction was stopped by cooling with a water bath to room temperature. The CsBr NCs were collected by centrifugation and redispersed in toluene-d_8.

Post-synthetic treatment

Size selection.[6] The fractional isolation of the supernatant proceeds through portion-wise anti-solvent addition (acetone), followed by centrifugation (29500 g at 17 °C, 10 minutes). The supernatant continues in the purification cycle, until no luminescence of the supernatant is observed, while the precipitate of each cycle constitutes an isolated fraction of NCs. These fractions are redispersed in toluene-d8 (0.5 mL).

Anion exchange. $CsPbBr_3$ and $CsPbCl_3$ NCs were mixed in several ratios (Table S2) of pure halide NCs in toluene-d8 to a final volume of 0.5 mL.

Table S2. Volumes of pure halide NCs used for the mixtures for the mixed halide NCs.

Ratio	$CsPbCl_3$ (mL)	$CsPbBr_3$ (mL)
0:100	0.000	0.500
5:95	0.025	0.475
25:75	0.125	0.375
50:50	0.250	0.250
75:25	0.375	0.125
95:5	0.475	0.025
100:0	0.500	0.000

Sensitivity Calculations

In this study, we posed the question whether solution NMR is prohibited also for $CsPbX_3$. We continued with a rough estimate for the relative signal intensity per measurement time for ^{133}Cs NMR vs. ^{77}Se and ^{113}Cd NMR, considering their receptivity at natural abundance relative to ^{1}H together with their typical T_1 relaxation times and FWHM in $CsPbBr_3$ and CdSe, respectively, determined by ssNMR (see Table S3), assuming similar linewidth and relaxation behavior for NCs compared to their bulk analogues. Based on these calculations, ^{133}Cs is expected to be more than 60 times more sensitive than ^{77}Se and ^{113}Cd. Based on the ^{77}Se data on CdSe NCs from Thayer et. al.,[7] a $CsPbBr_3$ NC sample concentration of 2.5 mg/mL would be required. We thus concluded that such solution NMR studies are fully feasible.

The sensitivity per time compared to ^{133}Cs were calculated with the following formula:

$$Sensitivity(X) = \frac{R^X}{R^{Cs}} \times \frac{2000\,Hz}{FWHM_X} \times \frac{109\,s}{T_{1,X}} \ ,$$

where 2000 Hz and 109 s are the general FWHM and T_1 relaxation time of ^{133}Cs in CsPbBr3 (see Table S3.)

Table S3. Calculated intensities per time for ^{77}Se and ^{113}Cd in CdSe compared to ^{133}Cs in $CsPbBr_3$.

Nucleus	Recept. vs. ^{1}H at nat. ab.	T_1 / s $^{(a)}$	FWHM / Hz $^{(b)}$	Sensitivity vs ^{133}Cs
^{77}Se	5,37E-4	30[8]	~5000[9]	0.016
^{113}Cd	0,00135	~120[8, 10]	3750[8]	0.014
^{133}Cs	0,0484	109[11]	~2000[12]	1
^{207}Pb	0.002	~1[13]	17600	0.512

$^{(a)}$ Values for bulk material due to the lack of NC values. (b) Values for NCs.

Thayer *et al.* used labelled ^{77}Se (61%) for their CdSe solution NMR study.[7] The concentration was around 40 mg in 0.25 mL. Compared to a 0.5 mL sample used for our study, this corresponds to 20.1 mg or 0.26 mmol of ^{77}Se. This would require 34.8 mg of ^{133}Cs, equal to 150 mg of $CsPbBr_3$. Including the increased sensitivity of ^{133}Cs calculated above, about 2.5 mg of $CsPbBr_3$ is expected to give similar intensity compared to the literature data on CdSe NCs. Unfortunately, no number of scans is mentioned for ^{77}Se, hampering a direct comparison.

Sample Characterizations

$CsPbX_3$ samples from Figure 1

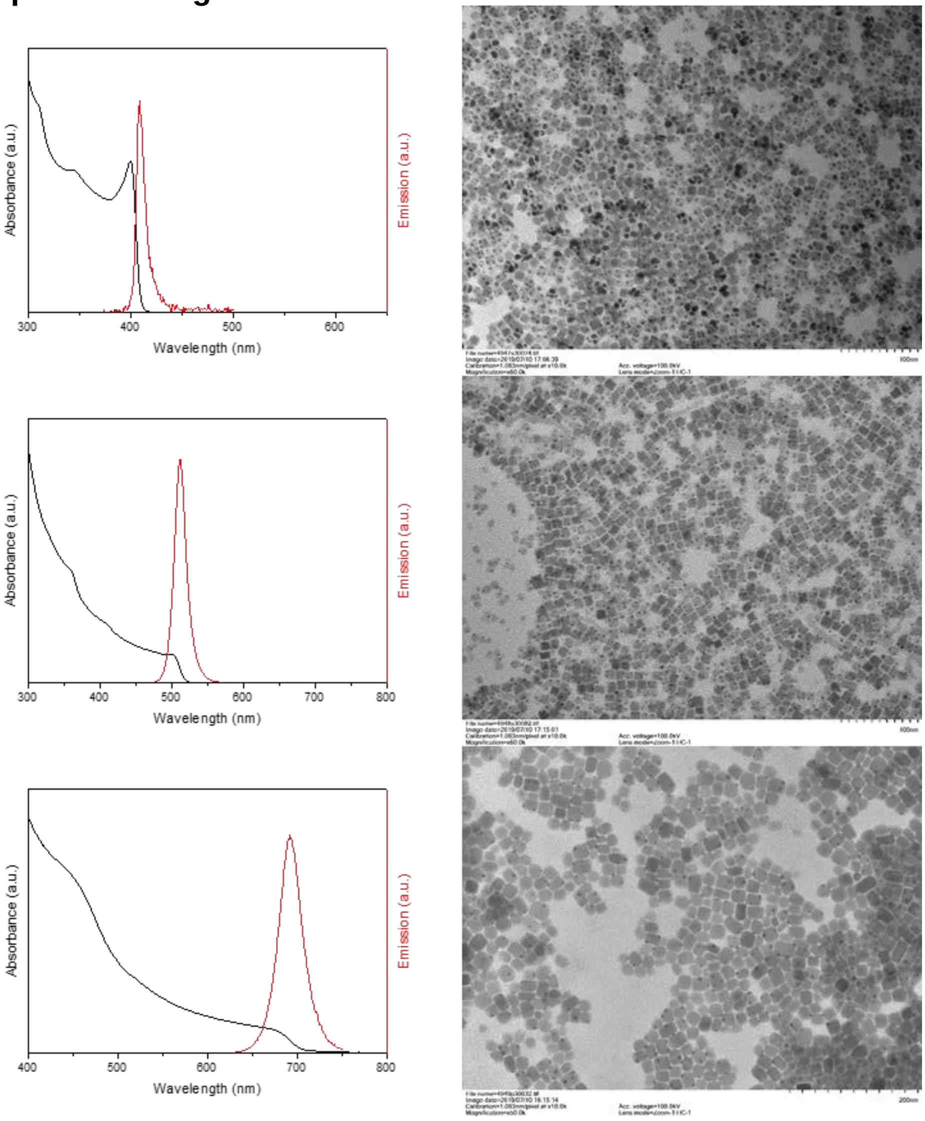

Figure S1. Absorption, PL and TEM o $CsPbX_3$ (X = Cl, Br or I from top to bottom) NCs.

Table S4. $CsPbX_3$ samples with their PL maximum, FWHM and the particle size determined by TEM.

Sample	PL (nm)	FWHM (nm)	Particle size (nm)
$CsPbCl_3$	408	10	10.1 ± 1.2
$CsPbBr_3$	512	21	10.3 ± 1.4
$CsPbI_3$	692	33	17 ± 3

Size selected lecithin-capped $CsPbBr_3$ NCs

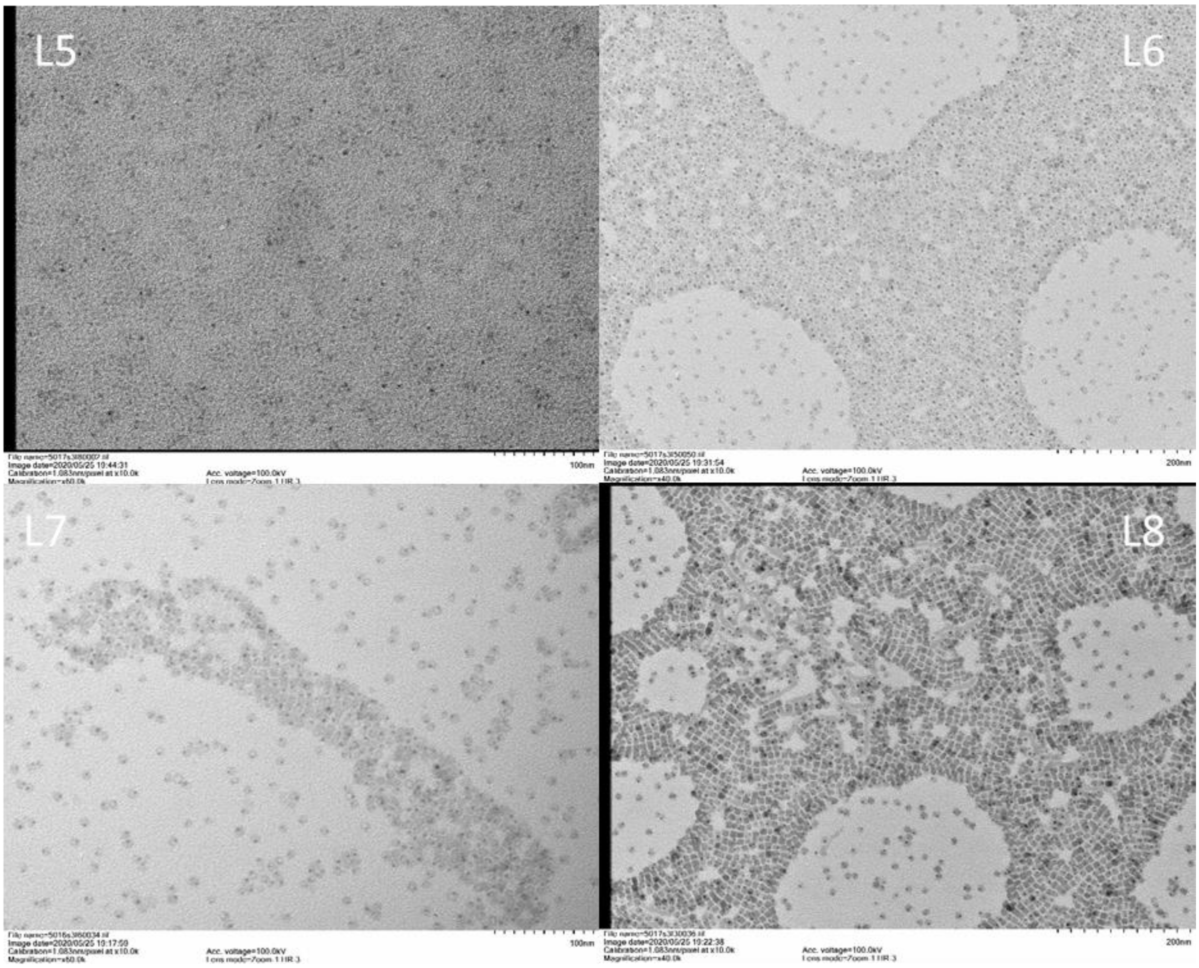

Figure S2. TEM images of the lecithin capped samples L5, L6, L7 and L8.

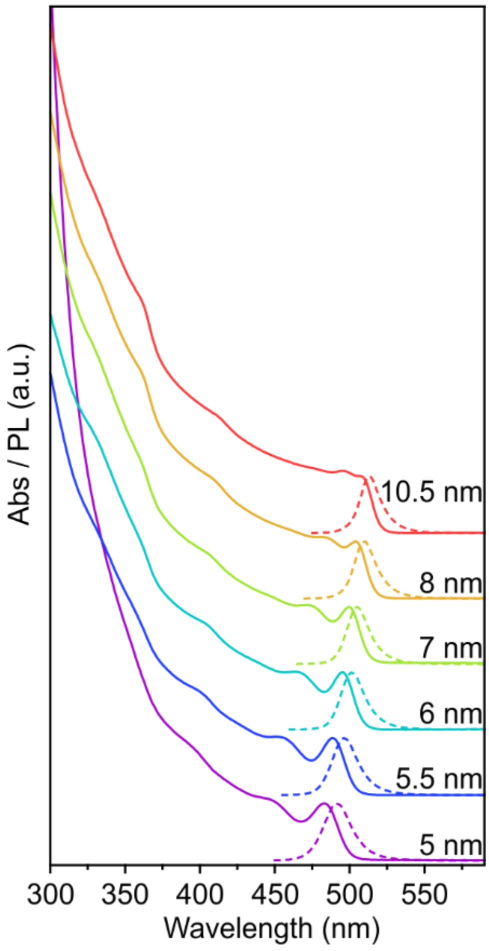

Figure S3. Absorption and PL spectra of size-selected fractions of lecithin-capped $CsPbBr_3$ NCs.

Table S5. PL, FWHM and particle sizes determined by a sizing curve and measured by TEM for size selected lecithin capped $CsPbBr_3$ NCs used for the size-dependance shown in Figure 2a.[6]

**Size / nm**	**PL / nm**	**FWHM / nm**	**Particle size / nm**		
			**Sizing curve**		**TEM**
			**c**	**a = b**	
5	491	22.2	4.7	5.6	-
5.5	496	20.1	5.0	6.0	-
6	501	18.8	5.6	6.7	6.2 ± 0.9
7	505	18.0	6.4	7.7	8.0 ± 0.9
8	510	18.1	7.3	8.8	7.5 ± 1.1
10.5	513	17.4	9.7	11.6	-
12	514	18.4	10	12.5	-

Size-selected ASC18-capped $CsPbBr_3$ NCs

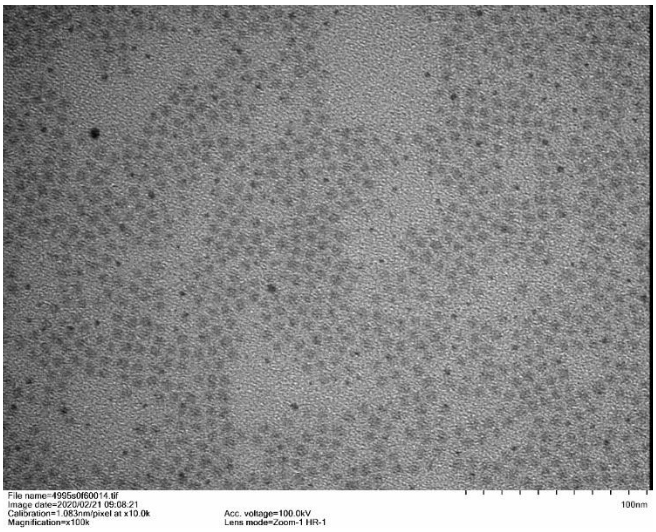

Figure S4. TEM picture of 4 nm ASC18 capped $CsPbBr_3$ NCs.

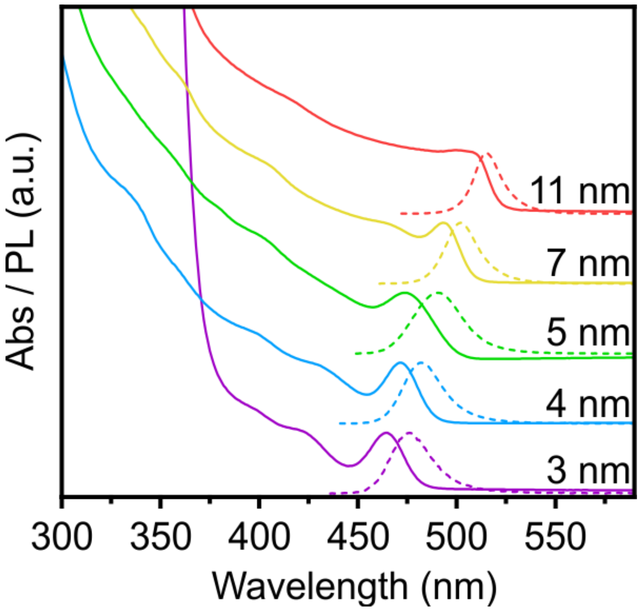

Figure S5. Absorption and PL spectra of size selected fractions of ASC18 capped $CsPbBr_3$ NCs.

Table S6. PL and FWHM for size selected ASC18 capped $CsPbBr_3$ NCs used for size-dependence shown in Figure 2e.

Size / nm	PL / nm	FWHM / nm
3	475	25
4	482	25
5	490	29
7	502	22
11	515	17

DDAB capped $CsPbBr_3$ NCs

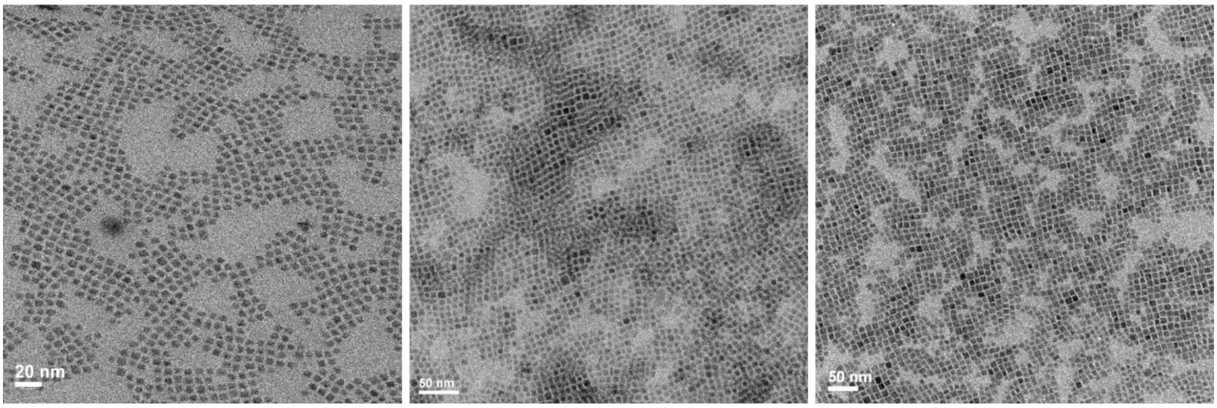

Figure S6. TEM picture of 5, 7 and 11 nm DDAB capped $CsPbBr_3$ NCs.

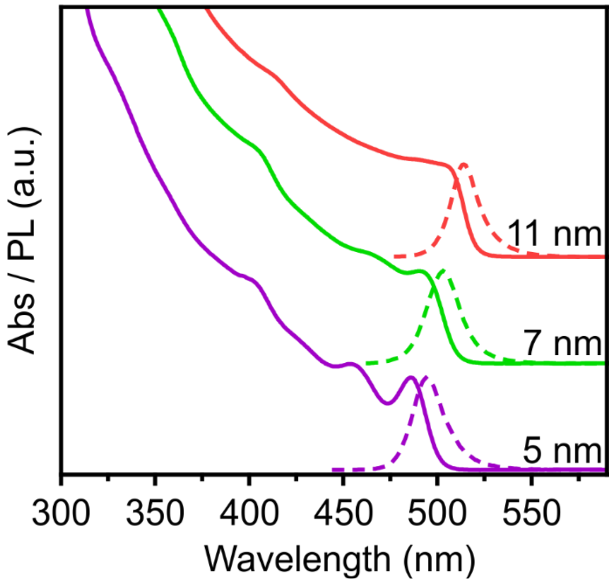

Figure S7. PL and absorption spectra of 5, 7 and 11 nm DDAB capped $CsPbBr_3$ NCs.

Table S7. PL and FWHM for DDAB capped $CsPbBr_3$ NCs used for size-dependence study shown in Figure 2i.

Sample	PL / nm	FWHM / nm
5	494	22
7	503	22
11	514	20

PPA-capped $CsPb(Br/I)_3$ NCs

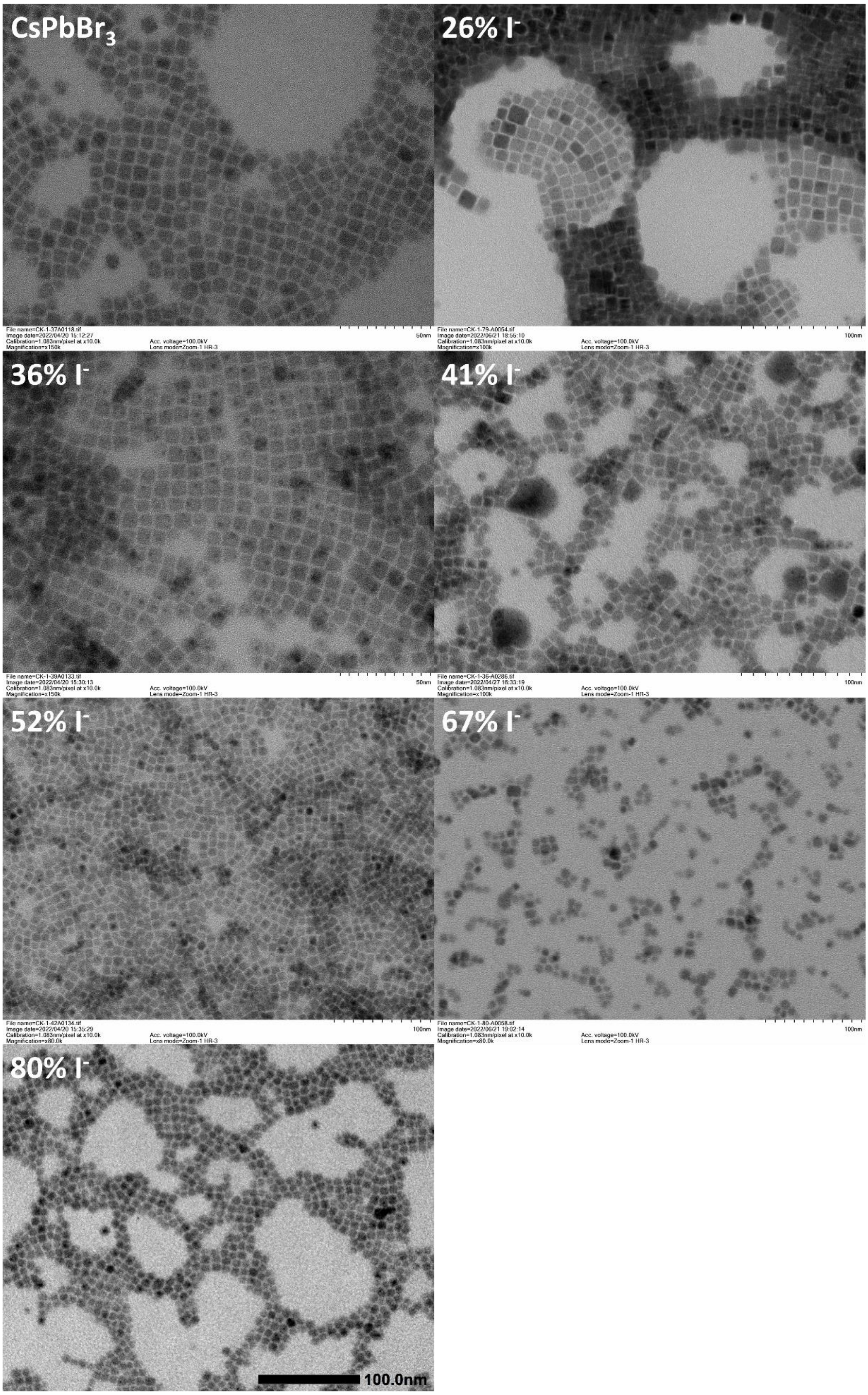

Figure S8. TEM images of PPA capped $CsPb(Br/I)_3$ NCs.

Table S8. PL, FWHM and size determined by TEM of PPA capped $CsPb(Br/I)_3$ NCs.

Sample	PL / nm	FWHM / nm	Size / nm
$CsPbBr_3$	503	19	7.1 ± 1.0
26% I^-	542	22	9.7 ± 1.8
36% I^-	553	28	8.4 ± 0.8
41% I^-	581	31	8.3 ± 1.3
52% I^-	600	34	8.3 ± 1.1
67% I^-	625	37	8.4 ± 1.3
80% I^-	642	33	7.9 ± 1.0

Table S9. EDX data of PPA capped $CsPb(Br/I)_3$ NCs. a) Only 1 sample was measured.

Sample	Synthesis I^- content / %	EDX I^- content / %
$CsPbBr_3$	0	1.7 ± - [a)]
26% I^-	29	26.3 ± 1.1
36% I^-	36	35.6 ± 2.3
41% I^-	43	41.1 ± 1.2
52% I^-	50	52.0 ± 2.1
67% I^-	57	67.1 ± 1.8
80% I^-	72	80.0 ± 4.0

PEA-capped $CsPb(Br/Cl)_3$ NCs

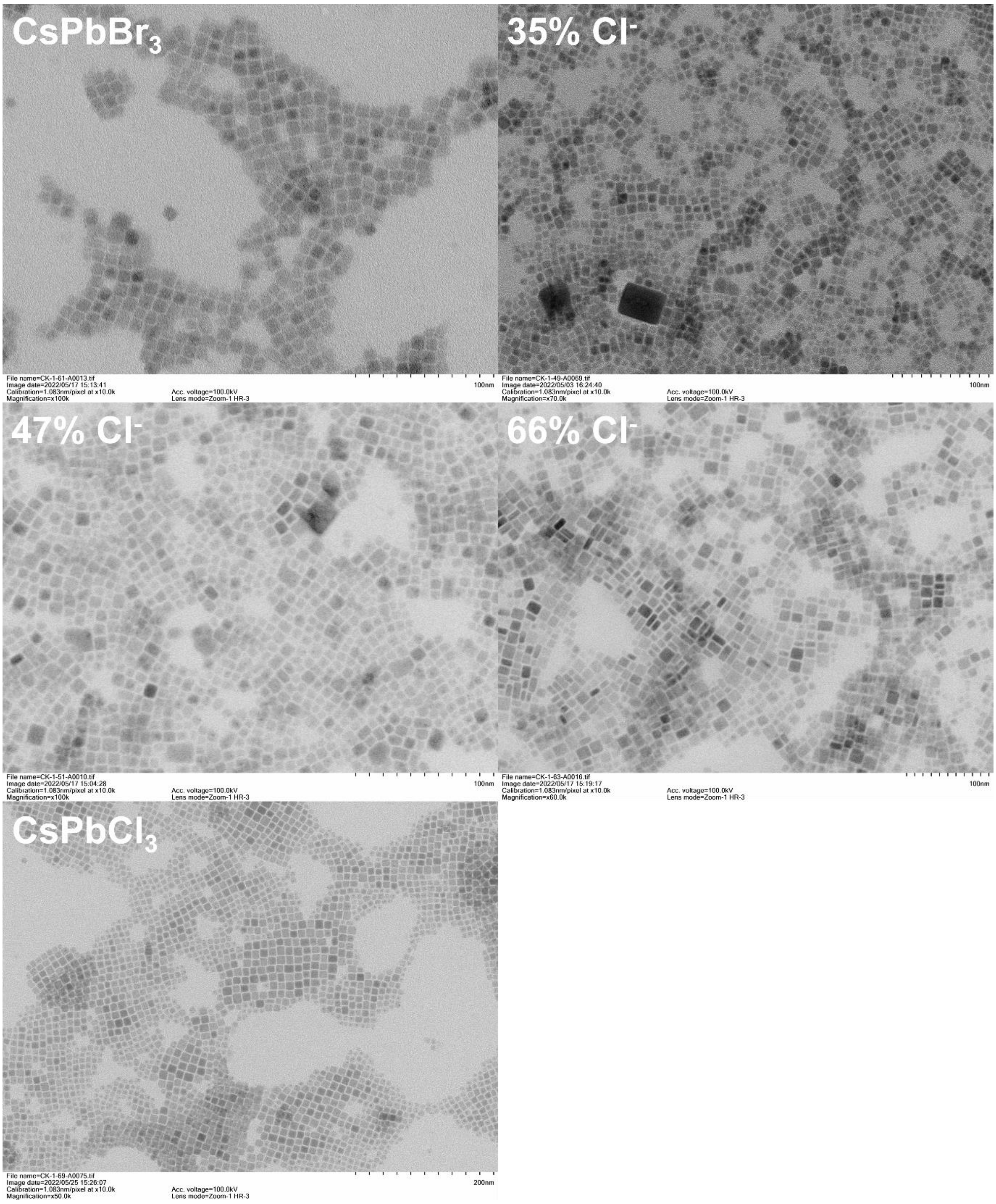

Figure S9. TEM images of PEA-capped $CsPb(Br/Cl)_3$ NCs.

Table S10. PL, FWHM and size determined by TEM of PEA-capped $CsPb(Br/Cl)_3$ NCs.

Sample	PL / nm	FWHM / nm	Size / nm
$CsPbBr_3$	509	16	9.3 ± 1.0
35% Cl^-	483	18	8.3 ± 1.7
46% Cl^-	470	19	7.7 ± 1.6
66% Cl^-	452	17	13.0 ± 3.0
$CsPbCl_3$	409	10	10.2 ± 1.4

Table S11. EDX data of PEA-capped $CsPb(Br/Cl)_3$ NCs. a) Only 1 sample was measured.

Sample	Synthesis Cl^- content / %	EDX Cl^- content / %
$CsPbBr_3$	0	-
35% Cl^-	19	34.5 ± 0.4
46% Cl^-	28	46.2 ± 1.0
66% Cl^-	43	66.4 ± 0.4
$CsPbCl_3$	100	100.0 ± - [a)]

Anion-exchanged lecithin-capped NCs

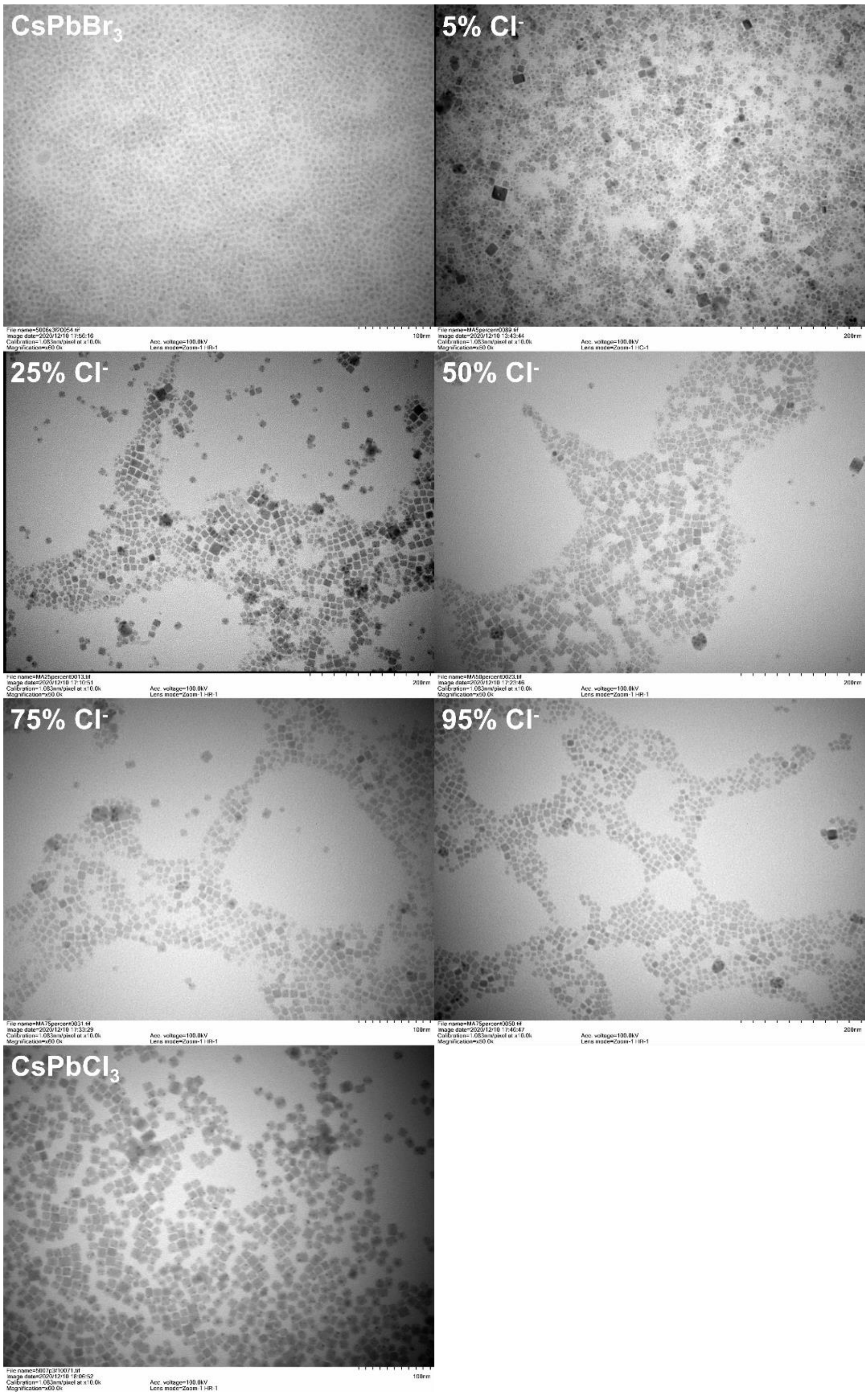

Figure S10. TEM images of lecithin capped $CsPb(Br/Cl)_3$ NCs.

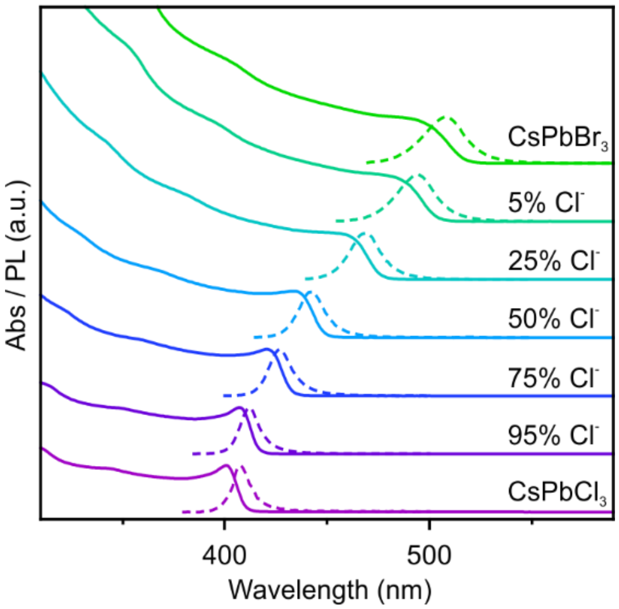

Figure S11. PL and absorption spectra of lecithin capped $CsPb(Br/Cl)_3$ NCs.

Table S12. PL, FWHM and size determined by TEM of lecithin capped $CsPb(Br/Cl)_3$ NCs.

Sample	PL / nm	FWHM / nm	Size / nm
$CsPbCl_3$	408	11	8.1 ± 1.1
95% Cl^-	412	12	9.4 ± 2.6
75% Cl^-	427	14	11.0 ± 3.2
50% Cl^-	442	15	9.4 ± 2.5
25% Cl^-	469	18	8.1 ± 1.8
5% Cl^-	494	23	8.8 ± 1.8
$CsPbBr_3$	508	26	8.5 ± 1.5

CsBr NCs

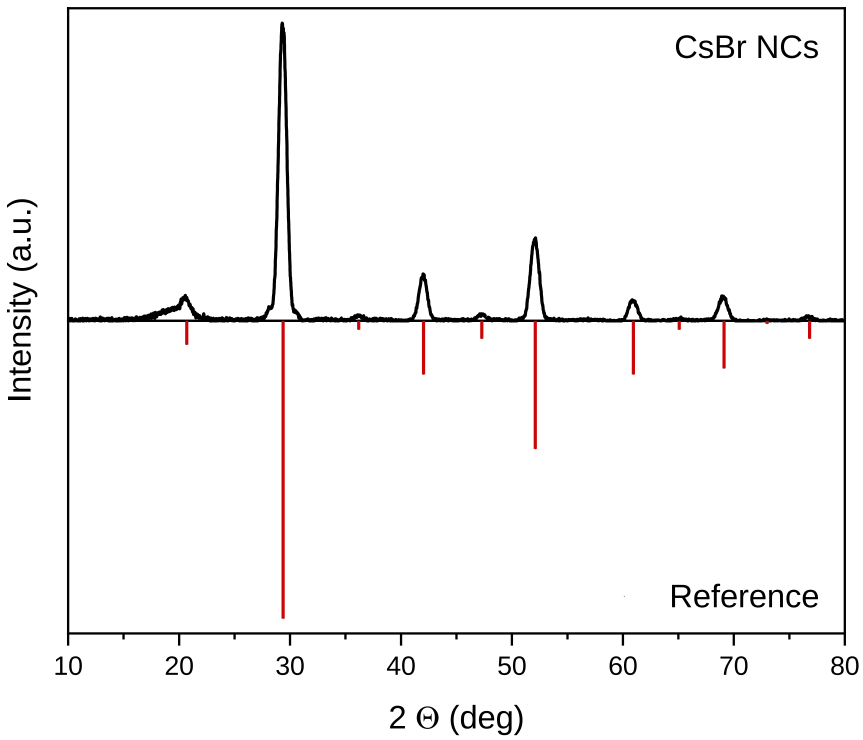

Figure S12. Powder XRD pattern of the CsBr NCs with the bulk reference pattern shown in negative.[14] The broad signal below 20° results from the adhesive tape used for the measurement.

Additional NMR data

Table S13. ^{133}Cs cNMR T_1 and T_2 relaxation times for $CsPbX_3$ NCs.

Ligand	Size / nm	Site	T_1 / s	T_2 / ms
Lecithin	5	Core	0.3	6.5
		Intermediate	0.2	0.8
		Surface	0.3	0.9
Lecithin	5.5	Core	1.2	5.0
		Intermediate	0.5	0.7
		Surface	0.5	0.7
Lecithin	6	Core	1.6	3.8
		Intermediate	1.6	1.9
		Surface	1.0	0.6
Lecithin	7	Core	1.8	2.8
		Intermediate	1.7	1.3
		Surface	1.5	0.6
Lecithin	8	Core	2.8	2.1
		Intermediate	2.7	1.0
		Surface	1.7	0.4
Lecithin	10.5	Core	3.5	2.0
		Intermediate	3.0	0.8
		Surface	2.5	0.2
ASC18	3	Core	-	-
		Intermediate	-	-
		Surface	0.3	1.8
ASC18	4	Core	2.6	5.2
		Intermediate	1.9	2.7
		Surface	0.4	0.9
ASC18	5	Core	1.5	8.2
		Intermediate	1.4	6.2
		Surface	0.3	0.7
ASC18	7	Core	1.8	5.7
		Intermediate	1.6	2.0
		Surface	0.3	0.5
ASC18	11	Core	-	-
		Intermediate	-	-
		Surface	-	-
DDAB	5	Core	1.0	11.5
		Intermediate	0.9	7.9
		Surface	0.7	2.4
DDAB	7	Core	1.3	9.7
		Intermediate	1.2	7.6
		Surface	1.0	1.7
DDAB	11	Core	2.4	1.5
		Intermediate	1.9	1.1
		Surface	0.9	0.5

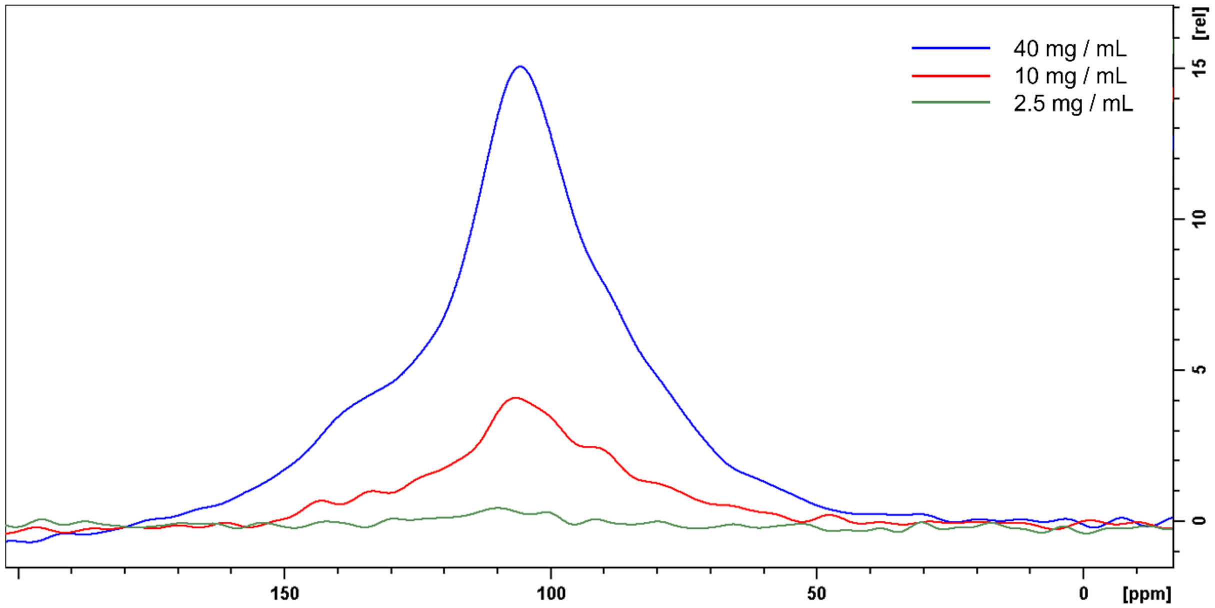

Figure S13. ^{133}Cs cNMR spectra of $CsPbBr_3$ NCs using various concentrations with the same amount of scans.

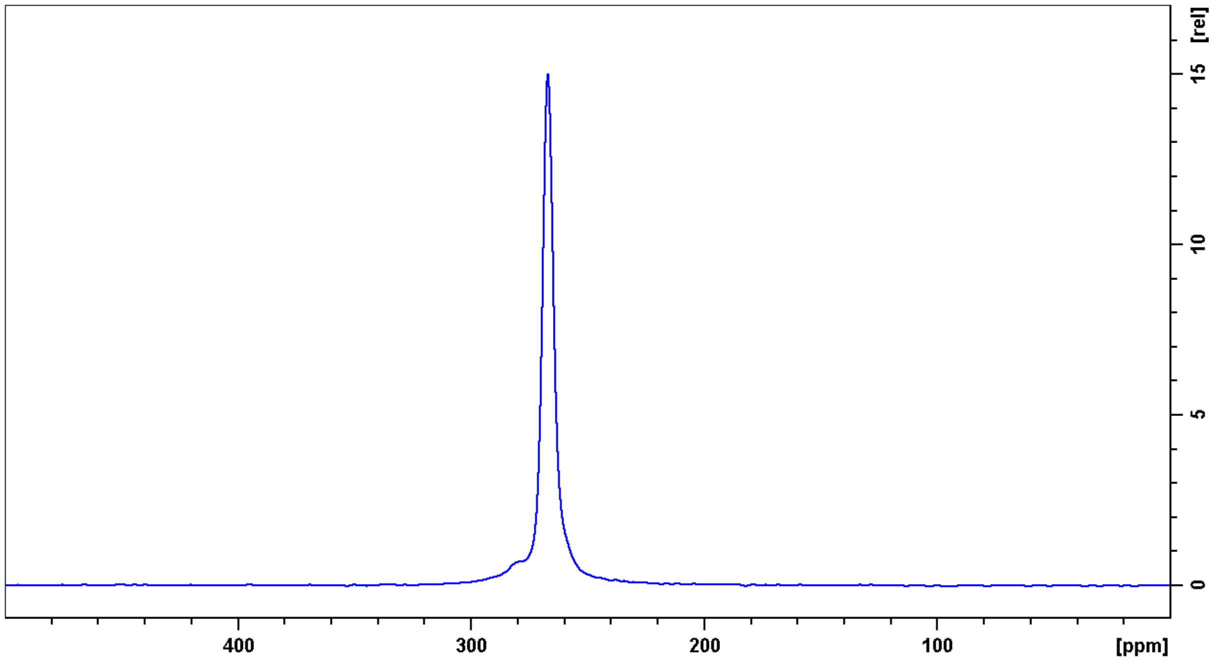

Figure S14. ^{133}Cs cNMR spectrum of CsBr NCs.

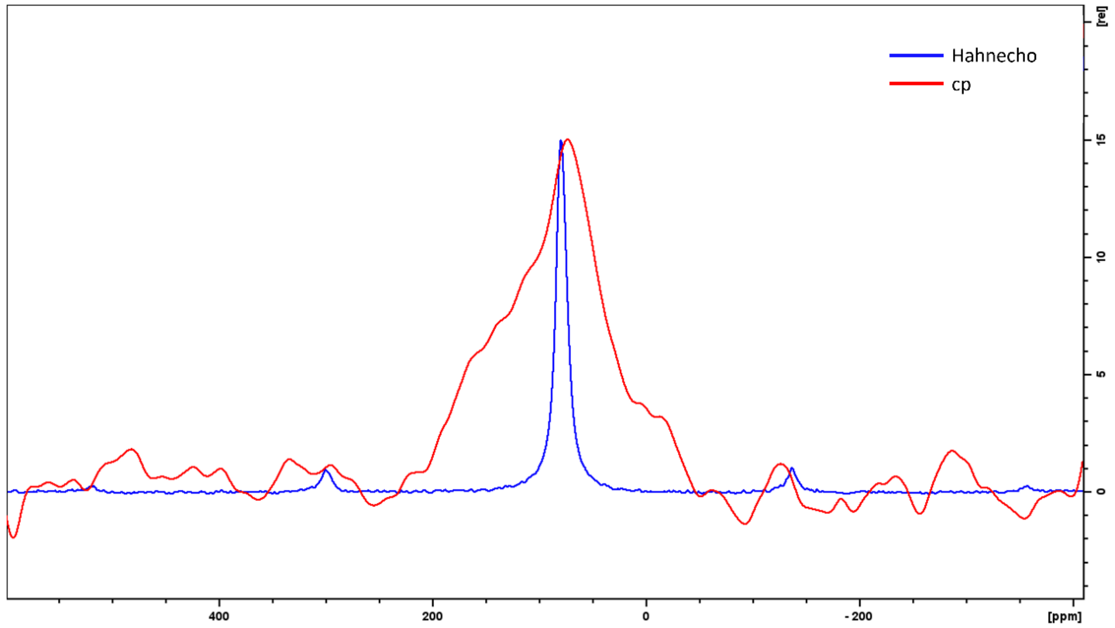

Figure S15. ^{133}Cs ssNMR spectra of $CsPbCl_3$ NCs acquired with a hahnecho sequence (blue) and with ^{1}H-^{133}Cs cross polarization (red) with a contact time of 2 ms.

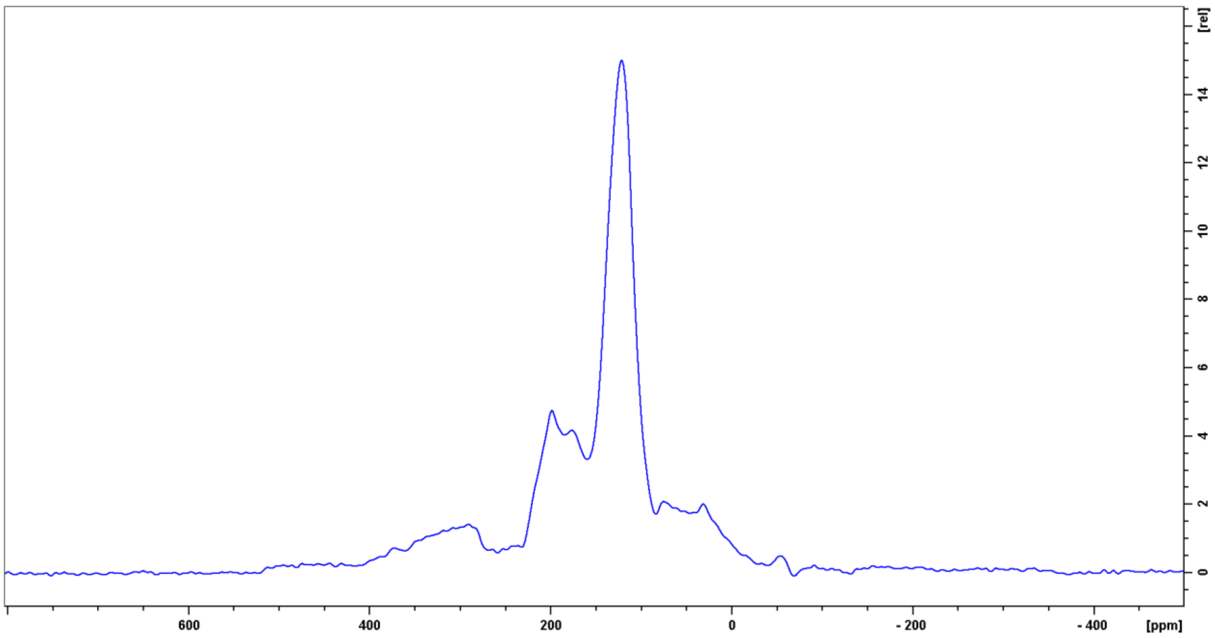

Figure S16. Static ^{133}Cs ssNMR spectrum of $CsPbBr_3$ bulk using a solution probe.

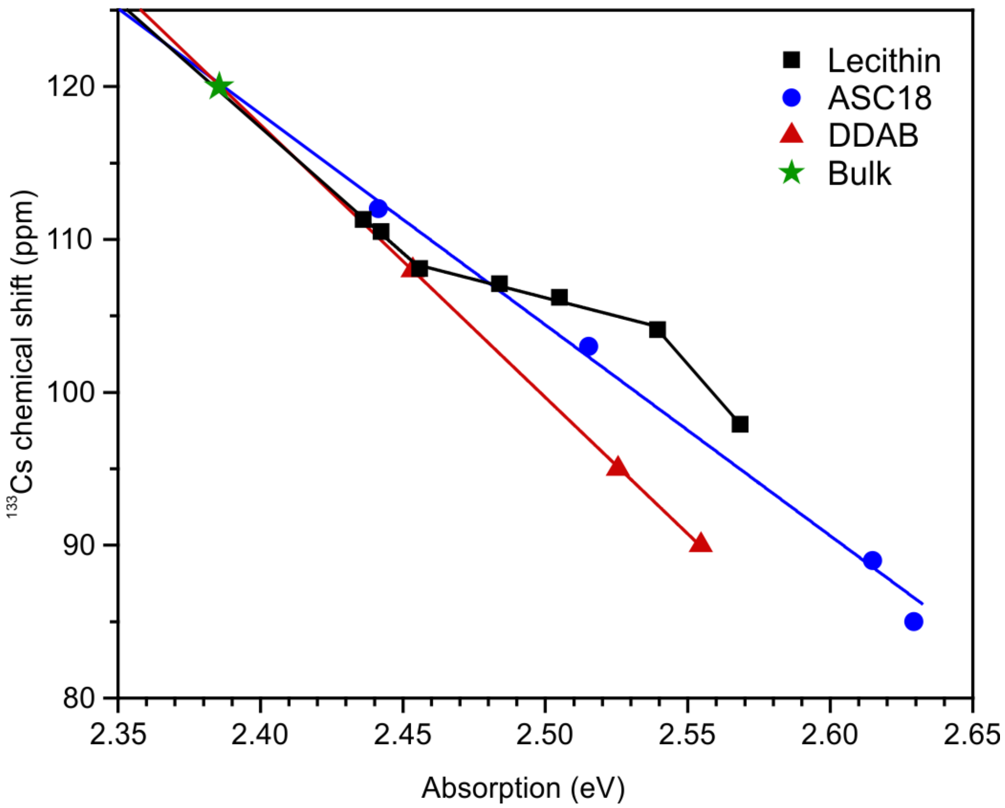

Figure S17. ^{133}Cs cNMR chemical shifts of the core peaks for monodisperse $CsPbBr_3$ NCs capped with various ligands versus their excitonic absorption energy.

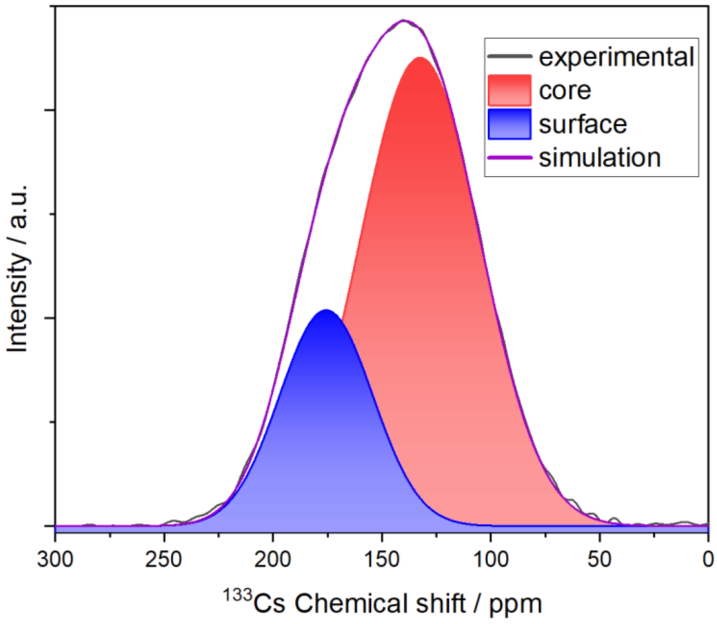

Figure S18. Fit of a ^{133}Cs cNMR spectrum of a $CsPb(Br/I)_3$ sample.

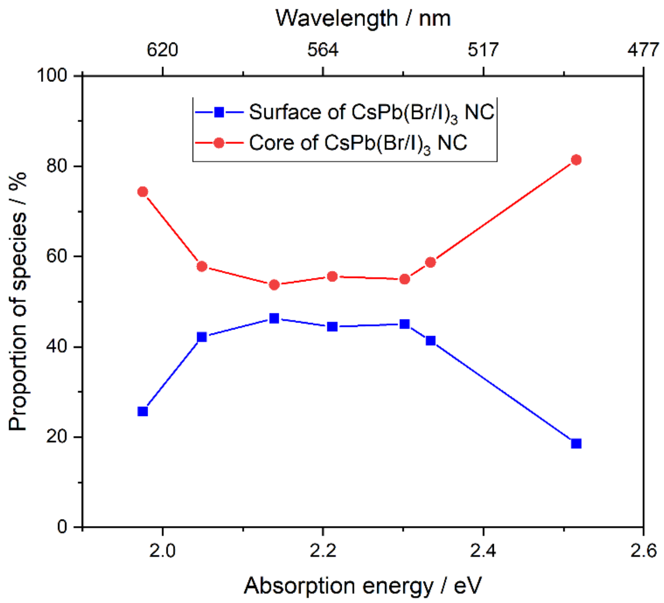

Figure S19. Surface and core contributions in mixed $CsPb(Br/I)_3$ NCs.

Additional DFT Figures

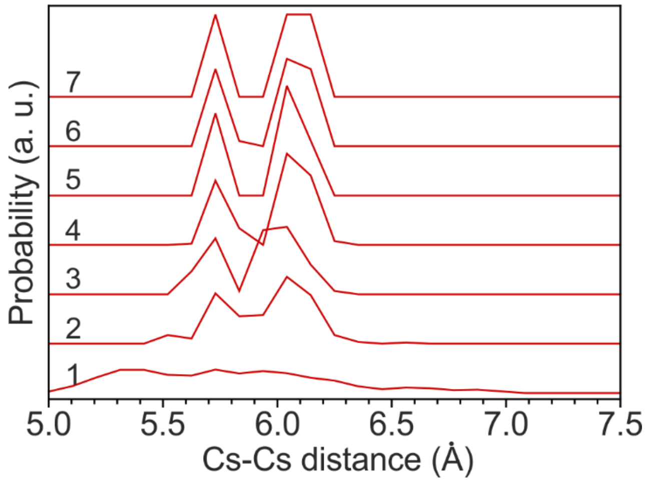

Figure S20. Cesium-cesium distances within a 5.4 nm $CsPbBr_3$ NC.

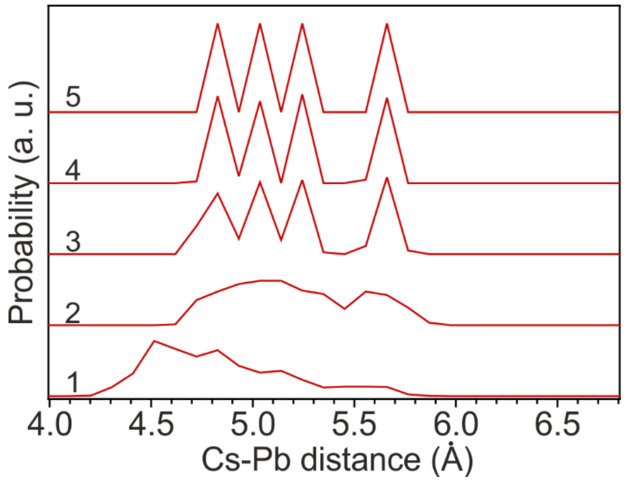

Figure S21. Cesium-lead distances within a 5.4 nm $CsPbBr_3$ NC.

References

(1) Krieg, F.; Ochsenbein, S. T.; Yakunin, S.; ten Brinck, S.; Aellen, P.; Süess, A.; Clerc, B.; Guggisberg, D.; Nazarenko, O.; Shynkarenko, Y.; et al. Colloidal $CsPbX_3$ (X = Cl, Br, I) Nanocrystals 2.0: Zwitterionic Capping Ligands for Improved Durability and Stability. *ACS Energy Lett.* **2018**, *3* (3), 641-646.
(2) Morad, V.; Stelmakh, A.; Svyrydenko, M.; Feld, L.; Aebli, M.; Affolter, J.; Kaul, C. J.; Schrenker, N. J.; Bals, S.; Sahin, Y.; et al. Designer Zwitterionic Phospholipid Capping Ligands for Structurally Soft Metal Halide Nanocrystals. *Nature* **2023**, 10.21203/rs.21203.rs-3595876/v3595871.
(3) Krieg, F.; Ong, Q. K.; Burian, M.; Rainò, G.; Naumenko, D.; Amenitsch, H.; Süess, A.; Grotevent, M. J.; Krumeich, F.; Bodnarchuk, M. I.; et al. Stable Ultraconcentrated and Ultradilute Colloids of $CsPbX_3$ (X = Cl, Br) Nanocrystals Using Natural Lecithin as a Capping Ligand. *J. Am. Chem. Soc.* **2019**, *141* (50), 19839-19849.
(4) Shynkarenko, Y.; Bodnarchuk, M. I.; Bernasconi, C.; Berezovska, Y.; Verteletskyi, V.; Ochsenbein, S. T.; Kovalenko, M. V. Direct Synthesis of Quaternary Alkylammonium-Capped Perovskite Nanocrystals for Efficient Blue and Green Light-Emitting Diodes. *ACS Energy Lett.* **2019**, *4* (11), 2703-2711.
(5) Shamsi, J.; Dang, Z.; Ijaz, P.; Abdelhady, A. L.; Bertoni, G.; Moreels, I.; Manna, L. Colloidal CsX (X = Cl, Br, I) Nanocrystals and Their Transformation to $CsPbX_3$ Nanocrystals by Cation Exchange. *Chem. Mater.* **2018**, *30* (1), 79-83.
(6) Krieg, F.; Sercel, P. C.; Burian, M.; Andrusiv, H.; Bodnarchuk, M. I.; Stöferle, T.; Mahrt, R. F.; Naumenko, D.; Amenitsch, H.; Rainò, G.; et al. Monodisperse Long-Chain Sulfobetaine-Capped $CsPbBr_3$ Nanocrystals and Their Superfluorescent Assemblies. *ACS Cent. Sci.* **2021**, *7* (1), 135-144.
(7) Thayer, A. M.; Steigerwald, M. L.; Duncan, T. M.; Douglass, D. C. NMR Study of Semiconductor Molecular Clusters. *Phys. Rev. Lett.* **1988**, *60* (25), 2673-2676.
(8) Ratcliffe, C. I.; Yu, K.; Ripmeester, J. A.; Badruz Zaman, M.; Badarau, C.; Singh, S. Solid state NMR studies of photoluminescent cadmium chalcogenide nanoparticles. *Physical Chemistry Chemical Physics* **2006**, *8* (30), 3510-3519.
(9) Piveteau, L.; Ong, T.-C.; Rossini, A. J.; Emsley, L.; Copéret, C.; Kovalenko, M. V. Structure of Colloidal Quantum Dots from Dynamic Nuclear Polarization Surface Enhanced NMR Spectroscopy. *Journal of the American Chemical Society* **2015**, *137* (43), 13964-13971.
(10) Xing, B.; Ge, S.; Zhao, J.; Yang, H.; Song, J.; Geng, Y.; Qiao, Y.; Gu, L.; Han, P.; Ma, G. Alloyed Crystalline CdSe1-xSx Semiconductive Nanomaterials – A Solid State 113Cd NMR Study. *ChemistryOpen* **2020**, *9* (10), 1018-1026.
(11) Kubicki, D. J.; Prochowicz, D.; Pinon, A.; Stevanato, G.; Hofstetter, A.; Zakeeruddin, S. M.; Grätzel, M.; Emsley, L. Doping and phase segregation in Mn2+- and Co2+-doped lead halide perovskites from 133Cs and 1H NMR relaxation enhancement. *Journal of Materials Chemistry A* **2019**, *7* (5), 2326-2333.
(12) Chen, Y.; Smock, S. R.; Flintgruber, A. H.; Perras, F. A.; Brutchey, R. L.; Rossini, A. J. Surface Termination of CsPbBr3 Perovskite Quantum Dots Determined by Solid-State NMR Spectroscopy. *Journal of the American Chemical Society* **2020**, *142* (13), 6117-6127.
(13) Aebli, M.; Piveteau, L.; Nazarenko, O.; Benin, B. M.; Krieg, F.; Verel, R.; Kovalenko, M. V. Lead-Halide Scalar Couplings in ^{207}Pb NMR of $APbX_3$ Perovskites (A = Cs, Methylammonium, Formamidinium; X = Cl, Br, I). *Sci. Rep.* **2020**, *10* (1), 8229.
(14) Swanson, S. E.; Gilfrich, N. T.; Ugrinic, G. M. *Standard X-ray Diffraction Powder Patterns*; U.S. Government Printing Office, 1954.
